# Sigma Hole Potentials as Tools: Quantifying and Partitioning Substituent Effects

**DOI:** 10.1021/acs.jpca.3c05797

**Published:** 2023-11-21

**Authors:** Kelling J. Donald, Nam Pham, Pranav Ravichandran

**Affiliations:** Department of Chemistry, Gottwald Center for the Sciences, University of Richmond, Richmond, Virginia 23173, United States

## Abstract

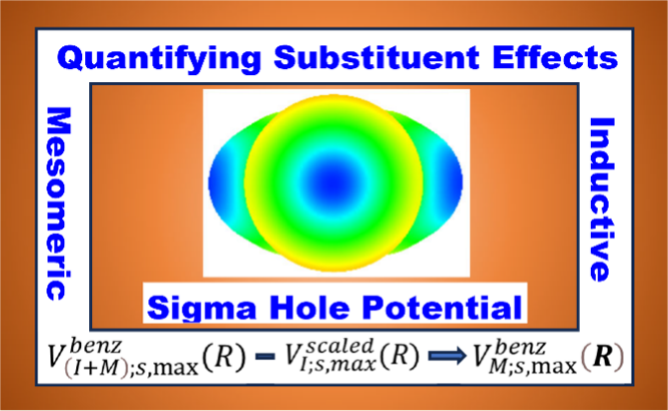

Empirical substituent constants, such as the Hammett parameters, have found important utility in organic and other areas of chemistry. They are useful both in predicting the impact of substitutions on chemical processes and in rationalizing after-the-fact observations on chemical bonding and reactivity. We assess the impact of substitutions on monoiodinated benzene rings and find that the modifications that substituents induce on the electrostatic potentials at the sigma hole on the terminal I center correlate strongly with established trends of common substituents. As an alternative to the experimental procedures involved in obtaining empirically based substituent constants, the computationally determined constants based on induced electrostatic potentials offer a model for quantifying the influence of mono- and polyatomic, neutral, and ionic substituents on their compounds. A partitioning scheme is proposed that allows us to discretely separate σ and π contributions to generate quantitative measures of substituent effects.

## Introduction

Almost 200 years after Faraday’s 1825 isolation and study of what is now called benzene (C_6_H_6_)^1,2^ and just over 150 years after Kekule reported his insightful model of its structure,^3−6^ the impact of substituents on the properties of that ring remains an area of active research in chemistry. This interest has persisted since substituted benzene rings are ubiquitous in modern chemistry and making a single substitution on the ring (C_6_H_5_R′) can radically modify the electron distribution in the ring and alter thus the likelihood that a subsequent substitution (to generate C_6_H_4_R′R′′) will occur at one or another of the remaining five C–H positions on the ring.

A given substituent R may operate as an electron donor or an acceptor via the σ skeleton and the π system of the ring and may further influence the electron density distribution in the molecules by field (short-range through-space polarization) interactions. To a first approximation (although the electron density distribution in the σ framework and in the π system are interrelated),^7^ the overall impacts of a substituent on the σ structure and on the π system, respectively, are summed up under the general categories of “inductive” and “mesomeric”^8^ effects (with the latter often conflated with ‘resonance’ or treated as a special case of it). The early trans-Atlantic contestation over the resonance and mesomerism concepts^9^ will not be rehashed here, but both terms remain in use, describing “resonance structures” for examples and “mesomeric effects”.

In addition to developing a qualitative understanding of the impact of substituents on acid ionization and reactivity of benzene derivatives, quantifying those substituent effects has been a major goal as well.^10−13^ Derick^14,15^ investigated the ‘Application of Polarity Measured in terms of a Logarithmic Functions of the Ionization Constant’ and the quantitative ‘Correlation of Ionization and Structure’^16,17^ and traced that effort even farther back to Ostwald.^14^ But it is Hammett’s culminating contributions, two decades later, to quantifying substituent effects that are the best known today.^18,19^ Hammett’s review of the field cited efforts by contemporaries to formulate a “definite and simple relation between the reaction rate and the free energy of dissociation [for acids].”^20^ But he was convinced that some of those efforts were “entirely incompetent,” and he made some key advancements.^18^

In brief, the general form of Hammett’s relationship linking the equilibrium constant, *K*, (or, alternatively, the rate constant, *k*) for the dissociation reaction of a substituted benzoic acid (R–C_6_H_4_–COOH) to the identity of the substituent, R, and its position on the ring is^19,21^
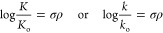
Here, *K*_o_ is the experimentally obtained equilibrium constant (*k*_0_ being the corresponding rate constants) for R = H, σ is a substituent constant specific to the identity of the substituent, and ρ is specific to the reaction, including the reaction medium and temperature. And Taft produced in the early 1950s a modified form of this equation to account more reliably for steric effects.^22−24^

Hammett-type constants have found extensive use in chemistry. They are guides, for instance, in tuning the reactivity of rings, rationalizing the impact of substituents on p*K*_a_ and reaction mechanisms, understanding weak interactions of rings,^25^ and even assessing trends in cocrystallization.^26^ And although the methods employed early on all relied on experimental quantities, some attention, however sporadic, has been given to computational approaches too for quantifying substituent effects.^27−30^ A 2005 review that is centered broadly on σ- and π-electron delocalization^28^ includes an account of some computational efforts made up to that point to link derived parameters (e.g., theoretical measures of aromaticity) and substituent constants. Substituent parameters based, for instance, on computed core–electron binding energies for ring carbon atoms^29^ and point charges^30^ have also been posited.

We develop here a rigorously defined and general computational descriptor for substituent effects that can be partitioned transparently into σ and π contributions. Our research group^31−33^ and others^34−36^ have considered that one viable computational approach for diagnosing the impact of a substitution, including inductive effects, on various properties of compounds is an assessment of the ensuing reorganization of the electron density distribution in the compound.

It is now well-known, for example (despite the expectation that a halogen atom, X, in a molecule, R–Y–X, is electron-rich), that a region of depleted electron density tends to arise on the outer pole of X opposite the Y–X bond, if R–Y is sufficiently electron-withdrawing.^37^ And that so-called sigma hole^38^ region is now a standard target of analysis for rationalizing halogen bonding.

That localized electron deficient region shows up as a maximum in the electrostatic potential (ESP) on the molecular surface, *V*_s_, and it tends to expand and become more positive as the “R–Y” fragment becomes more electron-withdrawing.^39^ In general, for a given “R–Y” fragment, the sigma hole on X becomes larger and *V*_s_ much more positive going down group 17 (from F to I), and it becomes increasingly feasible to form halogen bonds (R–Y–X ← base). Indeed, it is now commonly believed that many weak interactions,^40−45^ organic and otherwise, including certain interactions to central atoms, are fostered by the presence of sigma holes.^46−52^

We consider the potential utility of the variation in the electrostatic potential maximum, *V*_s,max_, at the sigma hole on iodine centers on substituted iodobenzenes, R–Ph–I, as a proxy for the *meta*- and *para*-directing influence of substituents on the ring. Bauzá et al. have examined the relationship between substituent constants and interaction energies of Y–I·N–Y′ complexes formed by the aromatic C_6_F_5_I species with certain *meta*- and *para*-substituted (pyridine, and cyanobenzene) bases.^34^ They identified ‘strong linear relationships’ between Hammett’s constants and interaction energies in those complexes and between those energies and the extremum values of the electrostatic potentials on I in the acids and N in the bases. Our results confirm a relationship between the potentials induced on I and the substituent effects for a broad range of substituents. We show the utility of computed potentials as alternatives for the traditional substituent constants, and importantly, a well-defined approach is introduced for partitioning the newly derived potential-based substituent parameters into σ and π contributions to the ring (de)activating tendencies of substituents. This approach allows for measures of (full, σ, and π) substituent effects to be computed as needed and compared for known or novel substituents.

## Computational Methods

The compounds considered in this work, including 62 iodobenzene systems and their saturated cyclohexane derivatives, have been optimized to minimum energy geometries on their respective potential energy surfaces and have been confirmed to be minima by vibrational frequency analyses (showing no imaginary frequencies). These computational studies have been carried out using the Gaussian 16 (G16) suite of programs,^53^ employing the ωB97X-D level of theory^54^ in combination with correlation-consistent triple-ζ (cc-pVTZ) basis sets^55^ for all atoms, except iodine, which is the heaviest atom in the compounds considered in this investigation. In the case of iodide, the small-core MDF pseudopotential^56^ provided by the Stuttgart/Cologne group and the associated triple-ζ basis sets^56^ were deployed. All calculations to examine the impact of solvent environments on the surface potentials of substituted benzenes were carried out using a self-consistent reaction field (SCRF) method. For all of the cases considered (for ethanol and water), we employed the polarizable continuum model (PCM).^57−60^ The Chemcraft^61^ and Gaussview 6^62^ graphical user interfaces have been used for data visualization and are the sources of molecular representations in this work. The electrostatic potential maxima presented herein were generated from formatted G16 checkpoint files using the Multiwfn software.^63,64^

## Results and Discussion

The substitution of a particularly electron-donating or -withdrawing substituent on a halobenzene (X-C_6_H_5_) ring can have a significant impact on the nature of the sigma hole on X in that molecule.^34^ For iodobenzene (where X = I), those effects are expected to be more prominent than they would be for any of the lighter halogen atoms due to the greater polarizability of iodine.^31,65^ And ipso facto, any interaction of bases with that X atom sigma hole is expected to strengthen as X gets larger, assuming, at least, that contact between the lone pair on the base and X is not frustrated, due to secondary (e.g., steric) effects such as the inconvenient presence of a bulky *ortho* substituent on an XC_6_H_5–*n*_R*_n_* ring^66^ or an inconvenient structural feature in a cumbersome base that limits the access of X to the lone pair.^67^ To avoid such scenarios in this work, therefore, and because of the well-established similarities in the electronic effects of *ortho*- and *para*-substitutions on the electron distribution in benzene rings, we consider in this contribution only *meta*- and *para*-substituted iodobenzenes. The magnitudes of the computed maximum electrostatic potentials (ESPs) in the sigma holes for all *meta-* and *para-*substituted iodobenzene systems considered in this work (62 molecules in total) are shown in Table 1.

**Table 1:** Computed Electrostatic Potential Maxima, *V*_s,max_, at the Sigma Hole on I in kcal·mol^–1^ Units (on the 0.001 au Isodensity Surface) of the R–C_6_H_4_–I Substituted Benzene Ring in the Gas Phase^a^

R	*meta*	*para*	R	*meta*	*para*	R	*meta*	*para*
H	17.67		B(OH)_3_^–^	–45.29	–40.93	CN	26.15	26.71
F	21.10	20.30	S^–^	–46.18	–43.47	CF_3_	23.22	23.66
Cl	21.76	21.54	NHBut	14.24	12.89	COOH	21.46	22.19
Br	21.86	21.70	NH_2_	15.37	14.01	COOCH_3_	19.46	21.02
I	21.53	21.60	NHCH_3_	14.66	13.17	NO_2_	26.29	27.37
CH_3_	16.68	16.49	NMe_2_	13.75	12.59	SO_3_H	25.98	26.52
CH_2_CH_3_	16.61	16.42	NHCHO	22.60	21.63	SO_2_Cl	27.91	29.05
*n*-Pr	16.24	16.04	OH	18.65	16.73	IF_4_	26.40	27.35
*i*-Pr	16.46	16.35	OCH_3_	16.39	15.92	ICl_2_	26.97	27.54
*t*-But	16.11	16.25	CHO	21.70	23.65	N≡N^+^	93.68	94.27
Ph	17.64	17.75						

a *i*-Pr: CH(CH_3_)_2_; *t*-But: C(CH_3_)_3_; these electrostatic potentials were generated on the 0.001 au surfaces. For R = I, the values are typically identical on both I centers. If they differ in any marginal way, the average values are used. ESP values are often reported in atomic units as well: 1 kcal·mol^–1^ = 1.5936 × 10^–3^ au.

**Figure 1:**
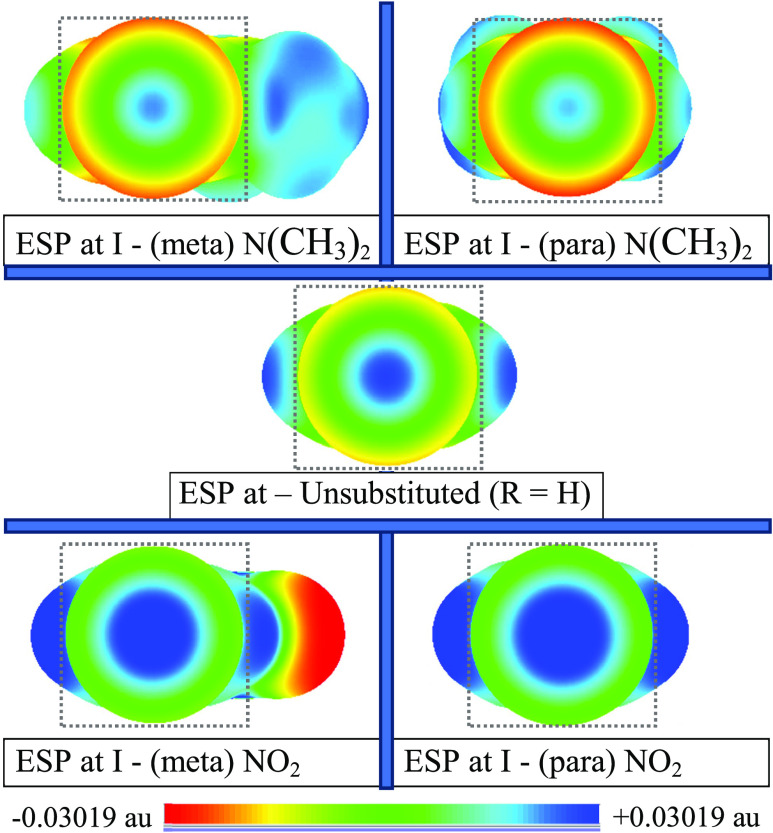
Electrostatic potential (ESP) maps showing the sigma hole on I (on the 0.001 au isodensity surface, all on the same ESP color scale: ±3.019 × 10^–2^ au) for R = H and inductively distinct substituents (R = N(CH_3_)_2_ and NO_2_) at *meta-* and *para-*positions in C_6_H_4_RI. Squares are added to help to identify the I site.

The substituents in Table 1 are grouped to reflect both the periodic relationships of the coordinating atoms (the first atom in each chemical formula in Table 1), as well as the generally understood (de)activating tendencies of the substituents. Representations of the computed electrostatic potentials on the surfaces of two pairs of *meta*- and *para*-substituted iodobenzene systems spanning the extremes for neutral donors and acceptors in Table 1 (R = N(CH_3_)_2_ and NO_2_) are shown in Figure 1. The potentials are all plotted on the same isodensity surface and on the same ESP color scale, and the two pairs of compounds are contrasted with the unsubstituted iodobenzene case (R = H), which happens to fall close to midway between them.

In each case in Figure 1, the structures are oriented horizontally (with R on the right of the ring in the *meta*-substituted species), with the iodine atom pointing out from the plane of the page and the sigma hole facing the reader. ESPs on the same color scale as that used in Figure 1 were generated for two of the ions that we have considered: R = S^–^ and N_2_^+^. But, in those more extreme cases (as we show in Table 1 and in the Supporting Information (SI); Figures S1–S2), the magnitudes of the electrostatic potential induced by –C_6_H_4_R on I for R = S^–^ and N_2_^+^ are very large (about an order of magnitude larger than those depicted in Figure 1). Indeed, |*V*_s_| on the 0.001 au surface is so large in those cases that on the color scale used in Figure 1, the ESP maps are totally and intensely red for S^–^, where *V*_s_ is uniformly negative and completely blue for N_2_^+^, where *V*_s_ is uniformly positive across the molecular surface (Figures S1–S2). Rescaled ESP maps for those two cases (on the same 0.001 au surface but using a larger, more sensitive range for the color scale) are provided in the SI as well (Figure S3). Although the surface potentials are all negative or positive for S^–^ and N_2_^+^, respectively, *V*_s_ does in fact vary from one point to another across the isodensity surface, and there is still a maximum (*V*_s,max_) at the I sigma hole in both cases (the least negative *V*_s_ on I for R = S^–^ and the most positive *V*_s_ on I for R = N_2_^+^), and those values are the *V*_s,max_ data shown in Table 1 for R = S^–^ and N_2_^+^ and similarly for B(OH)_3_^–^.

*V*_s,max_ is usually at the center of the sigma hole on the isodensity surface. The contrast in the size and strength of the iodine sigma hole (where “strength” is a loose term referring to how positive the *V*_s,max_ values are, which is indicated pictorially by the intensity of the blue region in Figure 1, for example) is indicative of the dramatic impact that a substituent (at either the *meta* or *para*) position can have on the sigma hole on a halogen atom substituent on a ring. The observation for both S^–^ and N_2_^+^—although they lie at the extremes of the potentials in Table 1—implies a definite relationship between the cumulative inductive and other electronic effects of substituents and the nature of the sigma hole on the halogen atom on the ring. More specifically (and in line with evidence provided elsewhere),^34,65^ substitutions at a given point on the ring may be used to tune or radically alter the potential at the sigma hole on X and, by extension, any halogen bond or other ESP-sensitive interaction in which a sigma hole might be involved.

**Figure 2:**
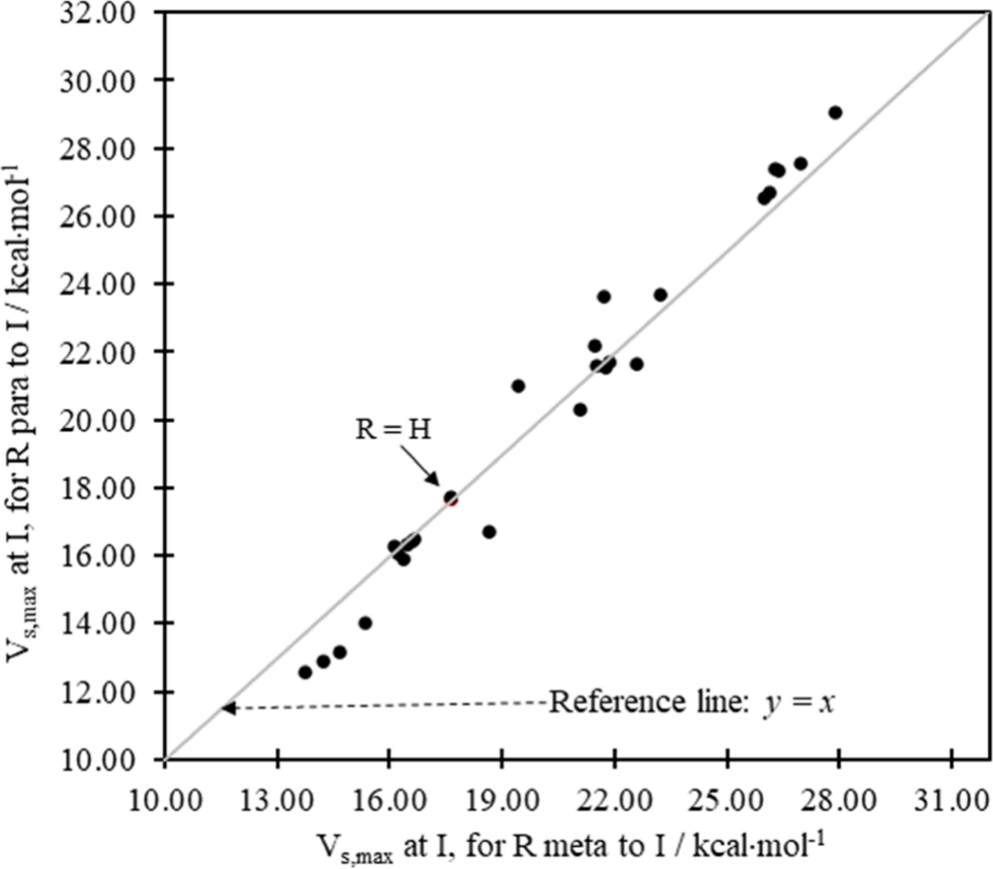
Plot of *V*_s,max_ in kcal·mol^–1^ at I for R at *para-* vs *meta*-positions on iodobenzene. All values are listed in Table 1. The (red) R = H data point is identified, partially obscured by R = Ph.

We will say much more shortly on the sensitivity of *V*_s,max_ at the sigma hole on I to the identity of the substituents in the gas phase, and in solution, but, to start with, the graphical representation of the two sets of data in Table 1 proves to be instructive (Figure 2). It provides us with insights, using the sigma hole as a sensor, into the electronic effects arising from changes in the substituent position on the ring.

The ESP maxima in the sigma hole at I in Table 1 for the *meta-* vs the *para*-positions relative to the position of I show a generally linear trend (see Figure 2). This implies that each substituent will produce about the same enhancement or attenuation of the sigma hole relative to the iodobenzene (R = H) regardless of the (*meta* vs *para*) position on the ring. But that is not quite the case. For reference, we include in Figure 2 and in other plots the line *y* = *x*. The case where R = H falls of necessity on that line since *V*_s,max(*meta*)_ = *V*_s,max(*para*)_ for R = H. In the idealized case, where mesomeric effects are negligible and the total field and inductive effects of substituents are independent of their positions on the ring, the induced ESP at I for any R at the *meta*- and *para*-positions should be identical such that all of the data points in Figure 2 would fall on the reference line *y* = *x*. But the distribution of the data (see Figures 2 and S4) is much more nuanced.

Several of the data points in Figure 2 do not fall on the *y* = *x* line, but they tend to cluster around it in a distinct pattern. For that reason, no best-fit line is provided, but the imposition of the reference line, *y* = *x*, is especially helpful since a close examination of the data evinces a link between the identity of R and the nature of the response of *V*_s,max_ at I to substituting for R.

**Figure 3:**
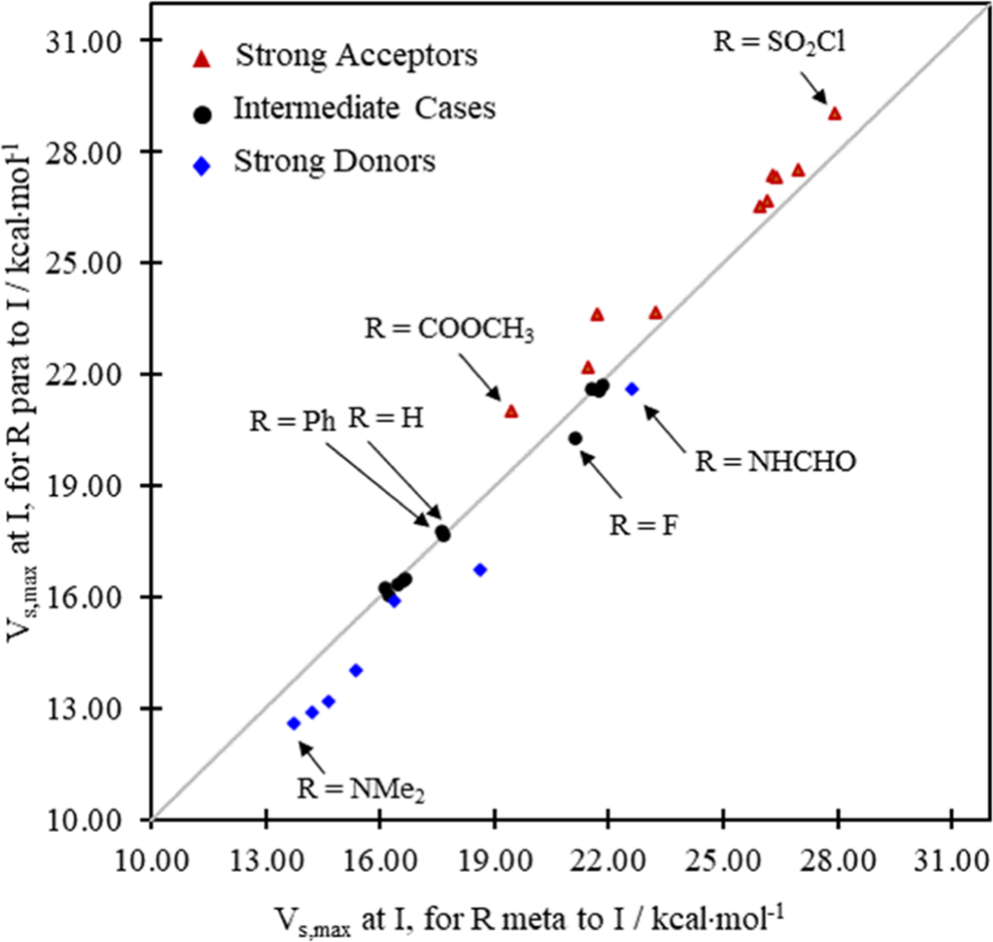
Plot of *V*_s,max_ in kcal·mol^–1^ at I for R at *para*- vs *meta-*positions on iodobenzene, exposing different responses of *V*_s,max_ as a function of the identity and position of R.

Figure 3 represents the data shown in Figure 2 in a way that amplifies certain distinguishing features of the computed potentials. The different marker types in Figure 3 help us to see that the three broad categories of substituents that we identified in Table 1 fall into definite subgroups as we go from the lower end to the upper end of the reference line on the graph.

For the weakest *V*_s,max_ values (blue diamonds in Figure 3)—where the potentials at the sigma hole on I tend to be noticeably lower (less positive) than *V*_s,max_ for R = H—we find that |*V*_s,max(*meta*)_| > |*V*_s,max(*para*)_|, that is, *para* substitution reduces the potential in the sigma hole even more substantially than *meta* substitution. Put another way, for this group of substituents, *para* substitution pushes more electron density into the ring, is more activating (toward electrophilic substitution), and diminishes, thus, *V*_s,max_ more strongly than *meta* substitution. There is an intermediate category of substituents (black circles) where |*V*_s,max(*meta*)_| ≈ |*V*_s,max(*para*)_|, and going up the reference line from left to right in Figure 3 (as *V*_s,max_ increases relative to the R = H case), we find a third subgroup of substituents (red triangles) for which *para* substitution increases the sigma hole potential more substantially than *meta* substitution, i.e., |*V*_s,max(*meta*)_| < |*V*_s,max(*para*)_|. For that group of substituents, *para* substitution pulls even more electron density from the ring, is more deactivating, and enhances, thus, *V*_s,max_ more strongly than *meta* substitution.

Moreover, it has been gratifying to find that the substituents in the three categories, respectively, fall roughly into electronically meaningful categories that we might describe as strong overall (i.e., σ + π) donors, intermediates, and strong overall (i.e., σ + π) acceptors. The species classified as strong donors are those in Table 1 from R = NHBut to OCH_3_ (blue diamonds in Figure 3). Several of them are already known to be good donors and typically have a lone pair of atoms bonded to the ring. Those classified as strong acceptors are the substituents in Table 1 from R = CHO to SO_2_Cl (red triangles in Figure 3), having, typically, very electronegative substituents (fluorides, such as CF_3_, and IF_4_, or double bonds to oxygen) on the central atom of the R group and no lone pair available to donate to the ring. The IF_4_ fragment, for example, is locally square pyramidal with a lone pair pointing away from the ring, opposite its C–I bond. ICl_2_ has two lone pairs, but in its T-shaped structure both lone pairs point away as well from the ring.

The systems described as intermediate cases include the halides, where strong σ-acceptor tendencies run counter to π donating tendencies, the phenyl ring ((R = Ph) which is traditionally considered to be weakly σ-withdrawing and weakly π-donating), and the alkyl substituents for which resonance or π contributions are expected to be weak relative to the stronger σ donor tendencies. And, except for the fluoride, these intermediate cases fall on or very close to the line *y* = *x* in Figure 3 with mean absolute percentage deviations in the *meta* and *para V*_s,max_ values in Table 1 of 1% or less. The well-known unique properties of the (very electronegative but π-donating) fluorine substituent likely account for their exceptional behavior compared to the other halogen atoms and the alkyl fragments in the intermediate group. A case may be made that F and Ph belong in another group, but we were content to leave them in that middle category for this discussion.

**Figure 4:**
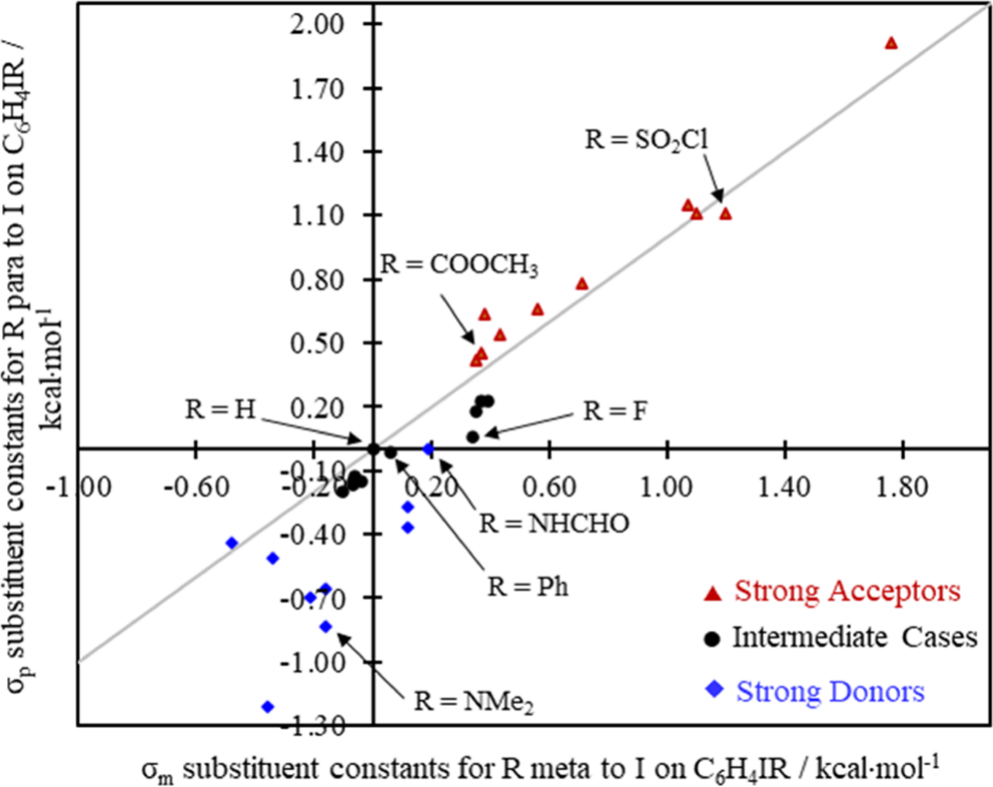
Plot of traditional substituent constants, σ_m_ and σ_p_, with distinguishing markers for the general categories of substituent types defined in Table 1.

Overall, the double (σ + π) donor systems fall consistently below the line *y* = *x*, the double (σ + π) acceptor systems fall above that reference line, and the intermediate systems tend to fall on or close to it (Figure 3). And that outcome provides us with some evidence that the electrostatic potentials induced at the sigma hole on the I center by the R group on the ring are potentially credible computational measures of the electron-withdrawing and -donating tendencies of substituents. Moreover, we find (as we show in Figure 4) that this is indeed the kind of general ordering that the classical (σ_m_ and σ_p_) substituent constants accomplish as well. There (in Figure 4) the donors (using identical labels to those used in Figure 3) assemble in the lower left quadrant of the graph, the intermediates in the middle, and the strong donors with their relatively large σ_m_ and σ_p_ values dominate the upper right. The values plotted in Figure 4 are listed in Table S1.^21,68^

Since the plot of the *para* vs *meta* effects on the I sigma hole succeeds in ordering the systems into categories as strong donors and acceptors (Figure 3; mirroring a pattern seen in the traditional constants (Figure 4)), we were encouraged to consider the extent to which these observations might allow us to rationally partition *V*_s,max_ into inductive and mesomeric components.

### ESP-Based Ansatz for Partitioning σ and π Contributions

We considered the possibility of partitioning the inductive and mesomeric contributions of the substituents by a scaling method that relies on the transferability of inductive effects from a six-membered-ring system without π-bonds. This approach has some precedence in a much earlier strategy to isolate inductive effects of substituents by assessing experimentally the reactivities of saturated systems—4-substituted bicyclo[2.2.2]octane-1-carboxylic acids—that (unlike substituted benzoic acid from which Hammett’s substituent constants were obtained) have no π-bonds.^69^

In this case, however, we considered the influence of equatorial substitutions at carbons 3 and 4 relative to the equatorial C–I bond in monoiodocyclohexane (C_6_H_10_RI), the saturated product of the *meta*- and *para*-substituted benzene systems that we have been discussing so far. The maximum induced electrostatic potentials at the sigma hole on I in each of the optimized benzene and saturated cyclohexane chair systems are listed in Tables S2 and S3 in the SI (and the corresponding minimum energy coordinates are available in the Supporting.xyz files). We will confirm later in this article a general insensitivity of the trends that we have observed so far to the equatorial vs axial position of iodine on the cyclohexane ring in generating transferrable inductive contributions to the overall value of the sigma hole potentials.

To compare the computed iodine sigma hole potentials from C_6_H_10_RI with the iodine sigma hole potentials obtained for the corresponding planar aromatic ring, the cyclohexane values were scaled according to the following simple ansatz:

(i) The maximum potential (*V*_s,max_) induced at the iodine σ-hole in C_6_H_10_RI due to field and inductive effects “I” of the ‘–C_6_H_10_R’ fragment (represented by *V*_I;s,max_^cycl^ (R)) is scaled by adding a constant Δ*V* to all of the *V*_I;s,max_^cycl^ (R) values. That constant is
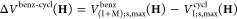
1It is defined to be precisely the difference between *V*_(I+M);s,max_^benz^ (**H**), which is *V*_s,max_ at I in iodobenzene (where R = H) and *V*_I;s,max_^cycl^ (**H**), which is *V*_s,max_ at I in iodocyclohexane (where R = H). So, the iodobenzene value and the scaled value are equal for R = H
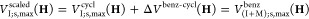
2Here, the implicit assumption is that for R = H, the substituent effects have no mesomeric contribution. And that same constant from R = H is added to all of the other cyclohexane values (see Tables S3 and S4) such that, in general, for any R, the scaled inductive term is
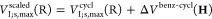
3

And, since *V*_I;s,max_^scaled^ excludes π effects for any given R group, we expect that generally
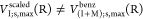
4except for R = H. Recall that, as defined above, the R = H case has no mesomeric component
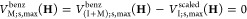
5

But for any arbitrary substituent, R, the corresponding mesomeric component
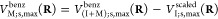
6is, in general, nonzero. The corresponding values, including those for *V*_I;s,max_^scaled^(R) and *V*_M;s,max_^benz^ (***R***), are shown in Tables S2–S5 in the SI. For an alternative and insightful route to the same definition for *V*_M;s,max_^benz^ (***R***), see the approach summarized in the Appendix section.

This scaling procedure provides us with transferable values associated with the specific field-inductive contribution of R to the total electrostatic potential at the sigma hole on I in benzene. It comes, however, with the assumptions that (a) the through bond electronic effect of R in C_6_H_10_RI is purely field-inductive and that through-space (field) effects of substituents at the *meta*- or *para*-position in the ring fall off rapidly with distance^69^ and (b) that an additive scaling strategy is valid for linking inductive potentials of the I center in the saturated ring and benzene.

**Figure 5:**
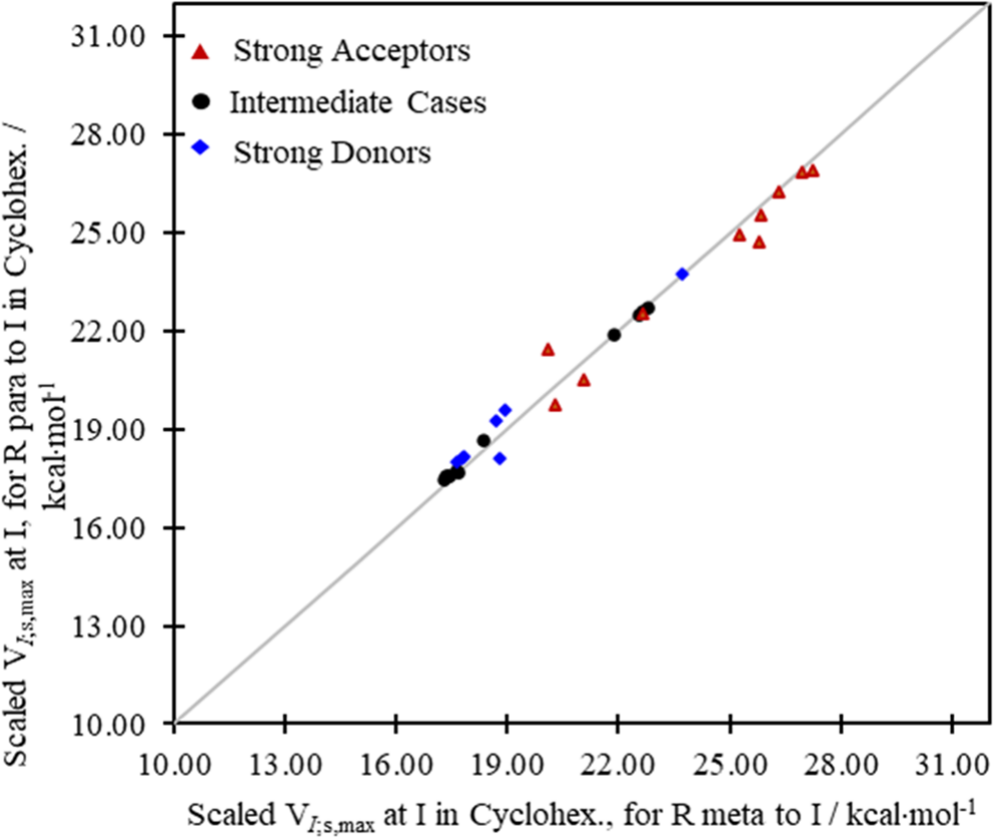
Scaled iodine σ-hole potential maxima obtained from cyclohexane and associated with field-inductive effects in the benzene ring for the *meta* and *para* R substituents.

The outcome for the scaled cyclohexane values for the *para-* vs *meta*-positions is shown in Figure 5. In that figure, the values all cluster very closely to the reference line, as expected for purely inductive effects. The position on the ring relative to the I center is expected indeed to be far less consequential for inductive effects that can be conveyed more evenly around the ring compared to the mesomeric effects^21^ such that *V*_I;s,max(*meta*)_ ≈ *V*_I;s,max(*para*)_. The complete graphs that include the ionic cases are included in the Supporting Information since the values for the charged species would compress the scale used here substantially (see Figures S4–S6 and Tables S2–S5).

**Figure 6:**
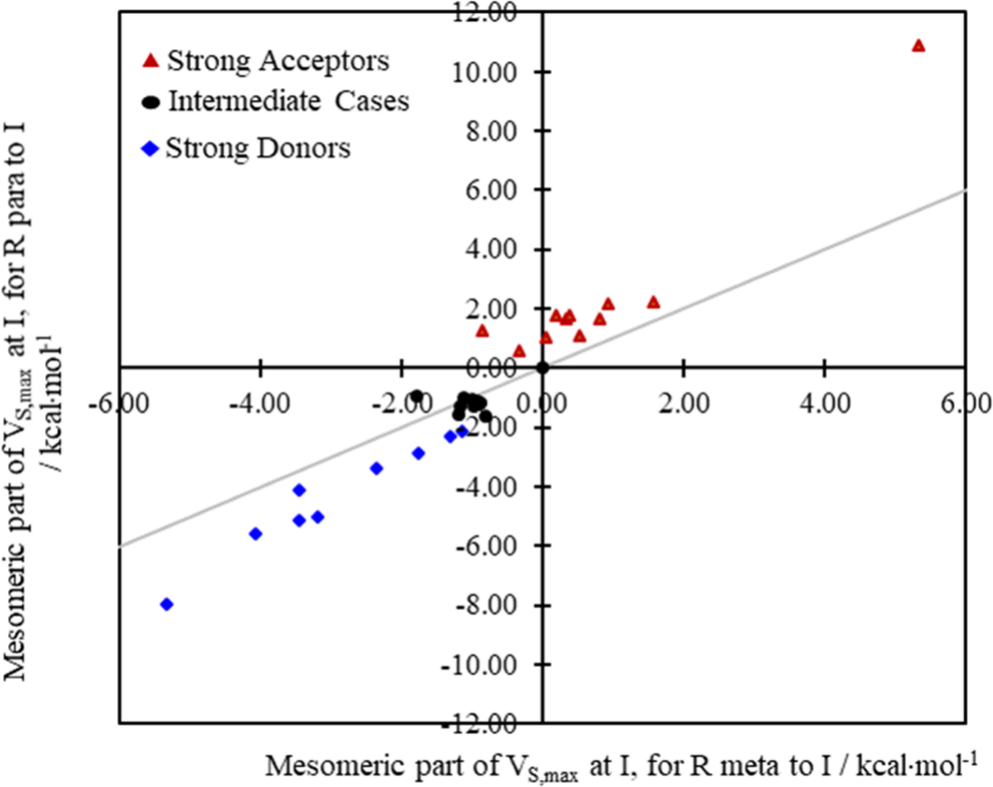
Plot of *para* vs *metaV*_M;s,max_^benz^ (R) values, which are associated with π effects after inductive components are removed from *V*_I+M;s,max_^benz^ (R).

By subtracting those scaled inductive contributions (Figure 5) from the corresponding total potentials (Figure 3), the partitioning strategy that we just outlined succeeds in isolating the strong π-donor systems (in blue, in Figure 6) from the strong π-acceptor systems (in red) to opposite sides of the reference line, with the intermediate systems falling on or very close to the line, including the R = H case where *V*_M;s,max_^benz^ = 0 by definition as we explained above.

Where R is a π-donor (and for *ortho*–*para* directors generally), the electron density in the ring is enhanced, including at I (and especially when R is at the *para*-position). So *V*_M;s,max_^benz^ (R) is expected to be lowered relative to *V*_M;s,max_^benz^ (H) = 0 for those systems, and therefore negative, as we observe in Figure 6 and Table S5. Since *V*_M;s,max_^benz^ (R) is the difference between the total *V*_(I+M)s,max_ at I and the putative inductive part, *V*_M;s,max_^benz^ exposes the deterioration of the sigma hole (due to the π donor’s infusion of electron density into the ring) relative to the R = H case.

Conversely, where I is a π-acceptor (and for *meta* directors generally), the electron density in the ring is diminished, including at I (and especially at the *ortho*- and *para*-positions) such that *V*_M;s,max_^benz^ (R) is expected to be increased relative to *V*_M;s,max_^benz^ (H) = 0 and are expected thus to be positive in general, as we observe in Figure 6 and Table S5. Since *V*_M;s,max_^benz^ (R) is the difference between the total *V*_(I+M)s,max_ at I and the putative inductive part, the *V*_M;s,max_^benz^ (R) values expose, in that case, the enhancement of the sigma hole (due to lower π electron density in the ring) relative to the R = H case.

The selection of the equatorial–equatorial (i.e., R_eq_–I_eq_) positions for I and R on the cyclohexane ring leaves unanswered the question of whether this outcome is an accident of our selection. We show in the SI (see the Supporting Notes and Figure S7) that indeed the general qualitative ordering of the substituents in terms of their inductive tendencies (and the impact on the sigma hole potentials) is not an accident of the C_6_H_10_RI configuration but is reflective of the nature of each R substituent.

### Modeling the Impact of Solvent Environments

**Figure 7:**
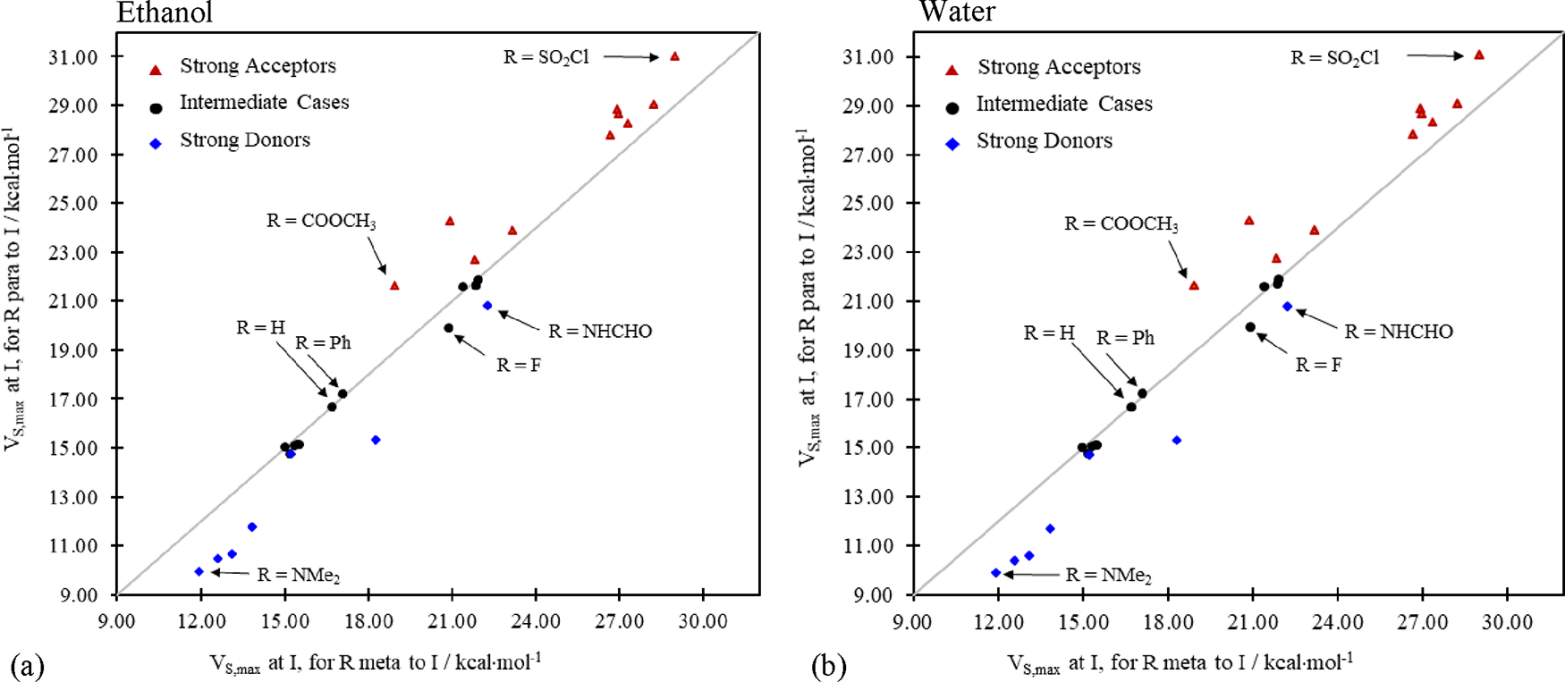
Plot of *para* vs *meta V*_(I+M);s,max_^benz^ values at I for (a) ethanol and (b) water. The values in the two graphs are very similar due to a rapid convergence of *V*_s,max_ with the relative permittivity, ε.

To model the influence of solvents on the strength of the sigma hole, we considered separately the two polar solvents (ethanol and water dielectric environments as defined by the implicit solvent PCM model in Gaussian 16) used in the solutions employed experimentally by Hammett.^18,19,21^ The analyses just reported for the gas-phase case were repeated, and those studies (see Figure 7 and Table 2) showed remarkable alignment with the data obtained from the gas-phase calculations (Figure 3), except that the actual magnitudes of the total *V*_s,max_ values are altered somewhat in those high dielectric environments, and we say more about that presently. An explicit solvent model affords vital insights where solvent–solute interactions are critical.^70,71^ We utilized an implicit solvent model, however, since, in addition to somewhat lower computational costs, the latter model allows us to assess the impact of substituents on the potentials in different dielectric environments (for the substituted benzenes and cyclohexanes (R–Y–I)), prior to any solute–solvent complex formation (e.g., R–Y–I·OEtH or R–Y–I·OH_2_ halogen bonds for water or ethanol, respectively). Such I·O type interactions are known to arise in solution^70^ and would necessarily inhibit our ability to locate and assess the isolated *V*_s,max_(I) (prior to any complex formation) in which we are interested here.

**Table 2:**
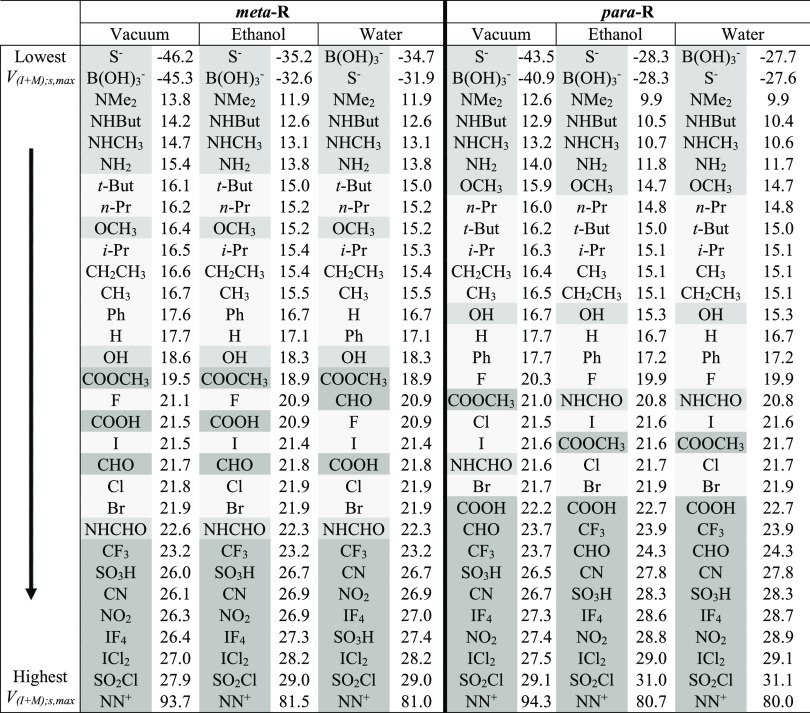
Total *V*_s,max_ at the I σ-Hole in C_6_H_4_RI, *V*_(I+M);s,max_^benz^, for Both the *meta*- and *para*-Positions in Different Solvent Environments

**Figure 8:**
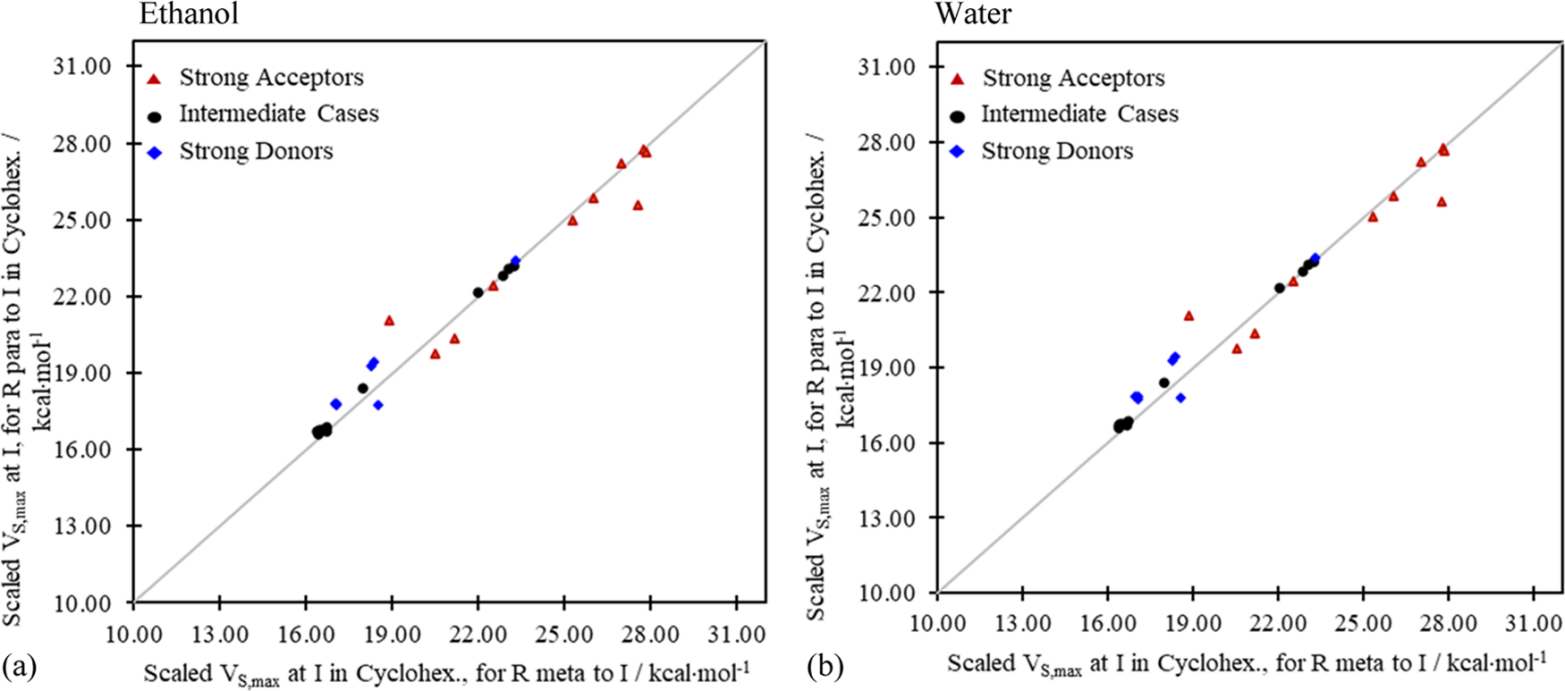
Scaled inductive potentials, *V*_I;s,max_^scaled^, at I for (a) ethanol and (b) water solvent environments. Identical trends are obtained for the unscaled *V*_I;s,max_^cycl^ before adding Δ*V*^benz-cycl^.

For iodobenzene, the computed potential maxima in the sigma hole at I, in kcal·mol^–1^ units, are *V*_s,max_ (R = H, vacuum) = 17.6, *V*_s,max_ (R = H, ethanol) = 16.7, and *V*_s,max_ (water) = 16.7. So, the solvent *V*_s,max_ values (listed in full in Table 2) agree for R = H up to three significant figures, which is in line with an earlier observation of an exponential convergence of ESP values as relative permittivity increases.^72^ That general qualitative agreement between results from the gas phase and from (implicit) solvent environments (Figures 3 and 7) extends to the isolated field-inductive terms as well. As we see in Figure 8, the computed scaled inductive potentials coalesce in general around the reference line *y* = *x* in all cases indicating little (*para* vs *meta*) position dependence of the trends in the inductive donating or withdrawing power of the substituents.

As we will show later in the article, results that are qualitatively very similar to those obtained from the gas phase (Figure 5) are obtained for the mesomeric components for the potentials in ethanol and water using the implicit solvent model described in the Computational Methods Section.

### Efficacy of Potentials for Quantifying Substituent Effects

A significant observation from the analysis so far is that the trends in the overall electron-withdrawing tendencies of the R groups considered, as expressed in the strengths of the sigma holes induced on I by ‘–C_6_H_4_R’, persist going from vacuum conditions to the (implict) solvent environments. In Table 2, the R groups are listed in order based on the value of the total maximum potential at the σ-hole on I in C_6_H_4_RI, that is, *V*_(I+M);s,max_^benz^. The values are shown for both the *meta*- and *para*-positions on benzene since the total effect of a given substituent can vary drastically with the position on the ring. Shading is used here to indicate the previously defined categories (Table 1) to which each R group was assigned.

The sensitivity of the induced electrostatic potentials to the position of R on the ring is evident in Table 2, for example, by shifts in the relative positions of the –NHCHO and −COOH groups in the *meta* vs the *para* columns for the three different conditions considered. Of note, the ordering appears to be less dependent on changes in ε (going across the three *meta* or the three *para* columns in Table 2) than they are on where R is on the ring. Notice, however, that the actual values of the potential maxima shrink by a few kcal·mol^–1^ (typically by much less than 10%) on going from the gas phase to ethanol and water in the implicit solvent models, except for the moderate to strong neutral acceptors (category 3 cases) at the bottom of the table. In those cases, the opposite response to the solvent environments is observed with the potentials increasing slightly.

Curiously, *V*_s,max_ shrinks as well in the solvent environments (Table 2) for both the positively and negatively charged species in the list. For those charged systems, the |*V*_s,max_| values are quite large relative to the neutral cases (see Table 2), and the percentage change in *V*_s,max_ relative to the gas phase, |Δ*V*_s,max_|, is somewhat larger as well (up to 35%). Since we only consider three instances of charged (anionic or cationic) R groups here, however, we refrain in this context from making any generalization on the response of such substituents to the chemical environment.

Since the *meta* and *para* values for the scaled field-inductive (*V*_I;s,max_^scaled^ (R)) potentials are rather close in value (cf. Figures 5 and 8), only the averages, [*V*_I;s,max_^scaled^ (R_*meta*_) + *V*_I;s,max_^scaled^ (R_*para*_)]/2, are presented in Table 3, but the full list of the individual *meta* and *para* values from which these averages are obtained are included in Table S6.

**Table 3:**
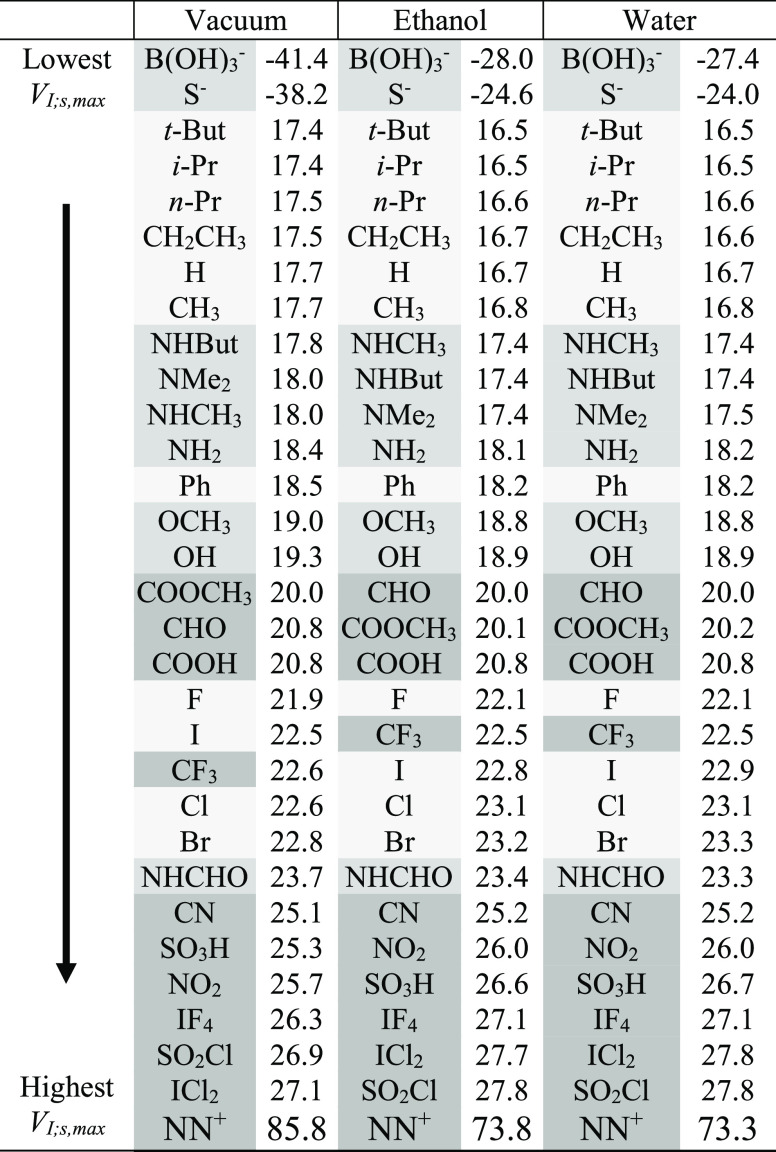
Average *V*_I;s,max_^*scaled*^ (R) Values for C_6_H_4_RI in Different Dielectric Environments

The averaged scaled field-inductive potentials (Table 3) show an ordering different from that observed for the total values in Table 2. The general separation of the (σ + π) donors and strong acceptors that is highlighted for the overall potentials in Table 2 is also observed for the purely inductive part (Table 3), but in the latter case, the alkyl substituents yield the lowest *V*_I;s,max_ potentials and are thus at the top of the table, that is, the alkyl groups, which are great σ-donors (species with substantial inductive *donor* effects), are the most successful R groups at inductively weakening the sigma hole on I relative to the unsubstituted C_6_H_11_I case. And, evidently, the alkyl groups lead to those low inductively induced *V*_I;s,max_ values on I in C_6_H_10_RI by donating so much electron density to the ring that (inductively, through the σ-framework) *V*_I;s,max_ is substantially attenuated relative to R = H (Table 3). The systems that are good (σ + π) donors, including the amines, appear after S^–^ and the alkyl groups, an indication that, without the π component, those R groups are really weakened as donors, becoming even weaker than the alkyl groups (hence their downward shift in Table 3 relative to that in Table 2). The halides, which are good inductive σ-acceptors (stripped in Table 3 of their counteracting π donor effects), appear even farther down in the columns in Table 3 among other strong inductive σ acceptors.

And what of the mesomeric contributions to the total potential under different dielectric environments? The component of the total sigma hole potential that is associated with mesomeric effects, *V*_M;s,max_^benz^, has been obtained as before (see Figure 6) using data that we generated under the specified solvent conditions by subtracting (see eq 6) the scaled inductive contributions from the total *V*_(I+M)s,max_ potentials (Table 4). For the sake of completeness, the gas-phase values (see Figure 6) are included in Table 4.

**Table 4:**
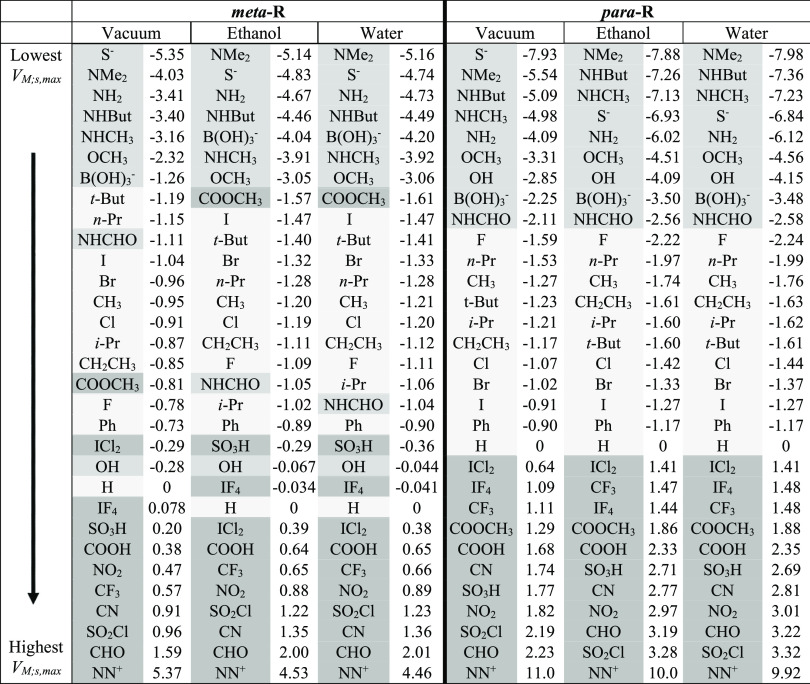
Resonance-Linked *V*_M;s,max_^benz^ (***R***) Values for C_6_H_4_RI in Different Dielectric Environments

Those outcomes are remarkably consistent with what we know about and expect classically from the π donors and acceptors that are considered here, signaling the substantial promise of terminal halogen sigma holes as tools in assessing the potential impact of novel substituent fragments on the π system of a ring and thus on the bonding in and reactivity of compounds.

For *para* substitution (see data on the right in Figure 4), the computed *V*_M;s,max_^benz^ values separate the (σ + π) donors and (σ + π) acceptors completely, with the intermediate cases sandwiched between them. That outcome is fully in line with the grouping of substituents adopted in Table 1 based on traditional assignments of the substituents as strong, weak, and intermediate (de)activating groups. And these results, consistent as they are with the experimentally rooted chemical intuition, suggest that the ESP analysis employed here may be readily applied to elucidate the influence of even less common or novel chemical substituents on aromatic rings.

The ordering is somewhat different for the *meta*-position, echoing a distinction between the influence of substituents in the *meta*-position versus the *para*-position that we saw in the unpartitioned total *V*_s,max_ values (Table 2) and which is a feature in the experimentally based Hammett substituent constants, σ_m_ and σ_p_ (Table S1).

Overall, a general increase is observed in the absolute value of the mesomeric component, |*V*_M;s,max_^benz^|, for neutral R groups going from the gas phase to polar solvents. So, the ansatz employed here suggests that π (accepting and donating) effects are enhanced in high dielectric environments, i.e., π donors are expected to become more successful at diminishing the strength of the sigma hole on I, and π acceptors will be more successful at accomplishing the reverse. Mesomeric effects influence the sigma hole indirectly, increasing (or decreasing) the electron density in the electron belt that surrounds the sigma hole on I, partially masking (or further exposing) the sigma hole as a consequence. Those π effects can be substantial, and they are recovered well by the partitioning scheme presented herein.

### Electrostatic Potentials as Alternative Measures of Substituent Effects

The computational derivation of substituent parameters that we have outlined in this work provides a new measure of the impact of substitutions on the electron distribution in compounds. The rigorously defined overall parameters are accessible at a low computational cost for substituents and may be employed in the interpretation of physicochemical properties of compounds and reaction processes analogous to experimental substituent constants. Beyond current computational approaches that propose descriptors for overall substituent effects or sigma constants only, for instance,^27−30^ a scheme is introduced here for partitioning the overall potential-based substituent parameters into distinct σ and π contributions. The provision of a partitioning framework is important since it provides a specific and rational basis for the systematic quantitative assessment and selection (from among known and potentially interesting novel options) substituents that are particularly suited for desired σ vs π electron-withdrawing and -donating tendencies.

## Summary and Outlook

A computational strategy is provided that allows us to examine the relationship between inductive and mesomeric influences of chemical substituents (on benzene) through an analysis of the induced potential at the sigma hole on a terminal atom on the ring. The investigation allows us to consider further as well the utility of induced electrostatic potentials as diagnostic tools in chemistry, even as we debate the role of electrostatics in accounting for weak interactions such as halogen bonding.^73^ We examine the influence of several mono- and polyatomic substituents, R, on the induced σ-holes on terminal I centers in substituted iodobenzene. The analysis allows us to probe and better understand the connection between inductive and mesomeric tendencies of substituents and the perturbation of the electron density in molecules and a few charged species.

A general correspondence is demonstrated between the computed potentials at sigma holes on I in substituted iodobenzene and classical empirical substituent constants. A readily implemented theoretical ansatz based on computed electrostatic potentials is proposed that partitions the potentials into reasonably well-defined categories as (σ and π) donors and acceptors.

The assumptions built into this model and its successes and limitations are discussed. The results emphasize the relevance of the π density in ring systems on σ-holes on terminal atoms. Donating electron density into the π system, for example, at both the *meta*- and the *para*-positions, leads to an evident expansion of the belt of electron density around the I nucleus perpendicular to the C–I bond in iodobenzene and an incipient contraction and weakening of the σ-hole. The σ-hole is π-dependent.

## Appendix: An Alternative Approach to Obtaining *V*_M;s,max_^benz^

Having computed *V*_(I+M);s,max_^benz^ (R) and *V*_I;s,max_^cycl^ (R), both sets of values may be scaled by subtraction to ensure that when R = H, *V*_(I+M);s,max_^benz^ (***H***) = 0, and *V*_I;s,max_^cycl^ (***H***) = 0. That is,

A1

A2

where the superscript ‘(*H*) = 0’ signifies that in both scaled data sets, *V*_s,max_(*H*) = 0. And, for any R,

A3

which will return exactly the *V*_M;s,max_^benz^ values given in the main text. An advantage to this approach is that it yields smaller numerical values for potential use as parameters for total, inductive, and mesomeric electronic effects (Table A1).

**Table A1:** Scaled (Gas Phase) ESP-Based *meta* and *para* Substituent Parameters, *V*_(I+M);s,max_^benz;(H) = 0^ (Total), and Their Inductive *V*_I;s,max_^cycl;(H) = 0^ and Mesomeric *V*_M;s,max_^benz^ Parts (See Figure S8)

	*meta*			*para*		
	total	I	M	total	I	M
H	0	0	0	0	0	0
F	3.43	4.20	–0.78	2.63	4.22	–1.59
Cl	4.09	5.00	–0.91	3.87	4.95	–1.07
Br	4.19	5.15	–0.96	4.03	5.05	–1.02
I	3.86	4.90	–1.04	3.93	4.84	–0.91
CH_3_	–0.99	–0.039	–0.95	–1.18	0.083	–1.27
CH_2_CH_3_	–1.06	–0.21	–0.85	–1.25	–0.081	–1.17
*n*-Pr	–1.43	–0.28	–1.15	–1.63	–0.10	–1.53
*i*-Pr	–1.21	–0.34	–0.87	–1.32	–0.11	–1.21
*t*-But	–1.56	–0.37	–1.19	–1.42	–0.19	–1.23
Ph	–0.029	0.70	–0.73	0.076	0.98	–0.90
B(OH)_3_^–^	–62.96	–61.71	–1.26	–58.60	–56.35	–2.25
S^–^	–63.85	–58.50	–5.35	–61.14	–53.21	–7.93
NHBut	–3.43	–0.026	–3.40	–4.78	0.31	–5.09
NH_2_	–2.30	1.11	–3.41	–3.66	0.43	–4.09
NHCH_3_	–3.01	0.15	–3.16	–4.50	0.49	–4.98
NMe_2_	–3.92	0.11	–4.03	–5.08	0.46	–5.54
NHCHO	4.93	6.04	–1.11	3.97	6.08	–2.11
OH	0.98	1.26	–0.28	–0.94	1.91	–2.85
OCH_3_	–1.28	1.03	–2.32	–1.75	1.57	–3.31
CHO	4.03	2.43	1.59	5.98	3.75	2.23
CN	8.48	7.57	0.91	9.04	7.29	1.74
CF3	5.55	4.99	0.57	5.99	4.89	1.11
COOH	3.79	3.40	0.38	4.52	2.84	1.68
COOCH_3_	1.79	2.60	–0.81	3.35	2.07	1.29
NO_2_	8.62	8.15	0.47	9.70	7.88	1.82
SO_3_H	8.31	8.12	0.20	8.85	7.07	1.77
SO_2_Cl	10.24	9.28	0.96	11.39	9.20	2.19
IF_4_	8.73	8.65	0.078	9.68	8.59	1.09
ICl_2_	9.30	9.59	–0.29	9.87	9.23	0.64
NN^+^	76.02	70.64	5.37	76.60	65.62	10.98

## Supporting Information Available

Additional notes; electrostatic potential maps for sample substituted iodobenzenes; plots of computed maximum potentials at the iodine sigma hole for *para*- and *meta*-substituted benzene and cyclohexane rings; computed maximum electrostatic potentials, and their partitioned inductive and mesomeric components (PDF)
xyz Files (ZIP)
