# Supplementary material for: Sigma Hole Potentials as Tools: Quantifying and Partitioning Substituent Effects
Source: J Phys Chem A. 2023 Nov 21;127(48):10147–58. doi: 10.1021/acs.jpca.3c05797 (PMC10711721; doi:10.1021/acs.jpca.3c05797)
Supplement: Supplementary file 1 — jp3c05797_si_001.pdf [file jp3c05797_si_001.pdf]

Sigma Hole Potentials as Tools: Quantifying and Partitioning Substituent Effects

Kelling J. Donald,* Nam Pham, and Pranav Ravichandran

Department of Chemistry, Gottwald Center for the Sciences, University of Richmond, Richmond, Virginia 23173, United States

Supporting Information

* Corresponding author. K. J. Donald, Tel.: 1-804-484-1628. E-mail: kdonald@richmond.edu ORCID: 0000-0001-9032-4225

Table of Contents

Abbreviated Captions*	Page
Guide to *.xyz* Files	S3
**Figure S1**: ESP maps showing the sigma hole on I in IC_6H_4R (without shadows on ESP maps), for sample R groups, including charged cases.	S4
**Figure S2**: ESP maps showing the sigma hole on I in IC_6H_4R (with shadows on ESP maps), for sample R groups, including charged cases.	S4
**Figure S3**: ESP maps on an expanded scale showing the sigma hole on I in IC_6H_4R (without and with shadows on ESP maps), for sample two charged cases.	S5
**Figure S4**: $V^{benz}_{(I+M);s,max}$: Para vs. meta plot of computed electrostatic potential (ESP) maxima at the I sigma hole, in kcal·mol^{-1} units, for I in IC_6H_4R.	S5
**Figure S5**: Para vs. meta plots of unscaled ($V^{cycl}_{I;s,\max}(R)$ (a)), and scaled ($V^{scaled}_{I;s,\max}(R)$ (b)) ESP maxima at the I sigma hole, in kcal·mol^{-1} units, for I on $IC_6H_{10}R$.	S6
**Figure S6**: Para vs. meta plot of $V^{benz}_{M;s,\max}(R)$	S6
**Supporting Notes 1:** On the consistency of computed potentials for different substitution patterns on cyclohexane.	S7
**Figure S7**: $V_{s,max}$ values for a diverse set of R substituents and $C_6H_{10}RI$ chair isomers.	S7
**Table S1**: Experiment based Hammett-type substituent constants	S8
**Table S2**: $V^{benz}_{(I+M);s,max}$: values for I sigma hole, in kcal·mol^{-1} units, for I in IC_6H_4R.	S8
**Table S3**: Unscaled ($V^{cycl}_{I;s,\max}$(R)) values.	S9
**Table S4**: Inductive $V^{scaled}_{I;s,\max}(R)$ values.	S9
**Table S5**: Mesomeric $V^{benz}_{M;s,\max}(R)$ values.	S9
**Table S6**: Unscaled and scaled inductive components for vacuum, and implicit solvent environments - ethanol and water.	S10
**Figure S8:** [Plots for scaling with $V_{s,max}$(H) = 0]: Para vs. meta plots for potentials at the sigma hole I on substituted benzene (a) and cyclohexane (b) molecules, with the values of the potentials adjusted so that $V_{s,max}$(H) = 0, and the mesomeric component (c).	S11
**Complete Gaussian 16 Reference**	S12

*More detailed captions are provided with the individual figures and tables.

Guide to *.xyz* Files: Coordinates of Structures Optimized and Examined

Cartesian Coordinates for key structures considered in this work are included as separate '.xyz' files (see the list below). The '.xyz' file format is readable by graphical user interfaces (GUIs) such as the Jmol and Chemcraft interfaces and in text format by editors such as Microsoft Word, WordPad, and Notepad++. All coordinates are for optimized geometries, each confirmed by vibrational frequency analyses to be a minimum on the potential energy surface.

#	Description of Coordinates in Supporting .xyz Files
**1**	(Gas Phase) I Meta to R on Benzene
**2**	(Gas Phase) I Para to R on Benzene
**3**	(Ethanol) I Meta to R on Benzene
**4**	(Ethanol) I Para to R on Benzene
**5**	(Water) I Meta to R on Benzene
**6**	(Water) I Para to R on Benzene
**7**	(Gas Phase) I Meta to R on Cyclohexane
**8**	(Gas Phase) I Para to R on Cyclohexane
**9**	(Ethanol) I Meta to R on Cyclohexane
**10**	(Ethanol) I Para to R on Cyclohexane
**11**	(Water) I Meta to R on Cyclohexane
**12**	(Water) I Para to R on Cyclohexane
**13**	(Gas Phase) Study of Different R-C_6H_{10}-I Isomers – R = H
**14**	(Gas Phase) Study of Different R-C_6H_{10}-I Isomers – R = F
**15**	(Gas Phase) Study of Different R-C_6H_{10}-I Isomers – R = Cl
**16**	(Gas Phase) Study of Different R-C_6H_{10}-I Isomers – R = Br
**17**	(Gas Phase) Study of Different R-C_6H_{10}-I Isomers – R = I
**18**	(Gas Phase) Study of Different R-C_6H_{10}-I Isomers – R = NH_2
**19**	(Gas Phase) Study of Different R-C_6H_{10}-I Isomers – R = CN

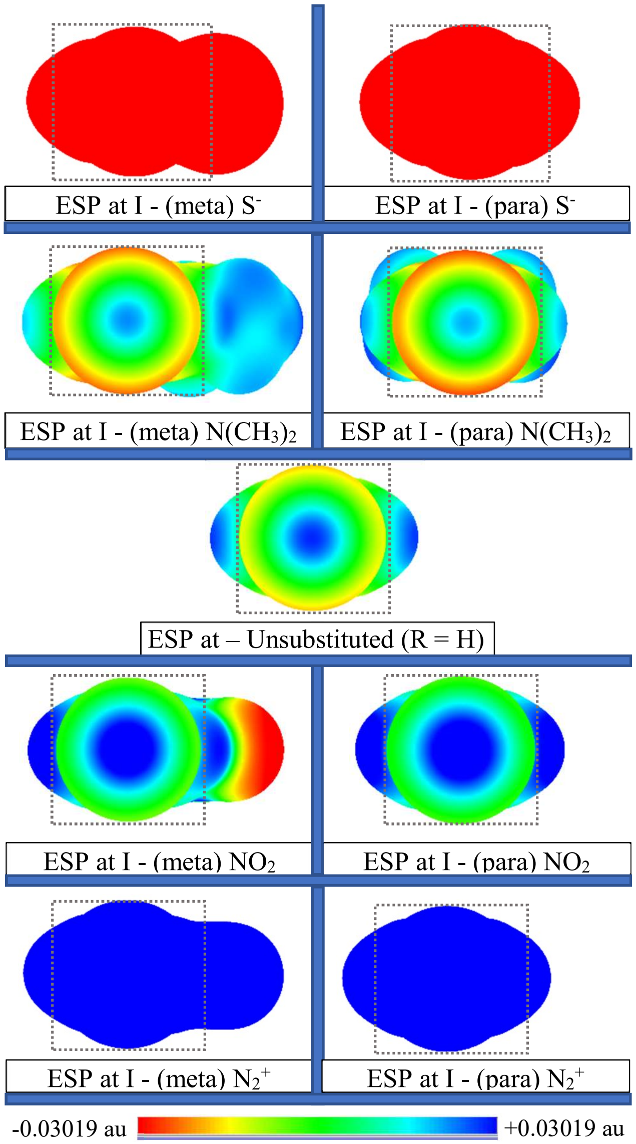

Figure S1: ESP maps showing the sigma hole on I (on the 0.001 au isodensity surface, all on the same scale: $\pm 3.019 \times 10^{-2}$ au.) for R = H and four other inductively distinct substituents (R = S^-, $N(CH_3)_2$, NO_2, and N_2^+) at both *meta* and *para* positions in C_6H_4RI. _For clarity, the ESP maps are shown without shadows_. Squares help to identify the I site. All structures are oriented with the ring horizontal and with I pointing out of the plane of the page. In the *meta* substituted species (on the left of this figure), the substituent is on the right of the ring.

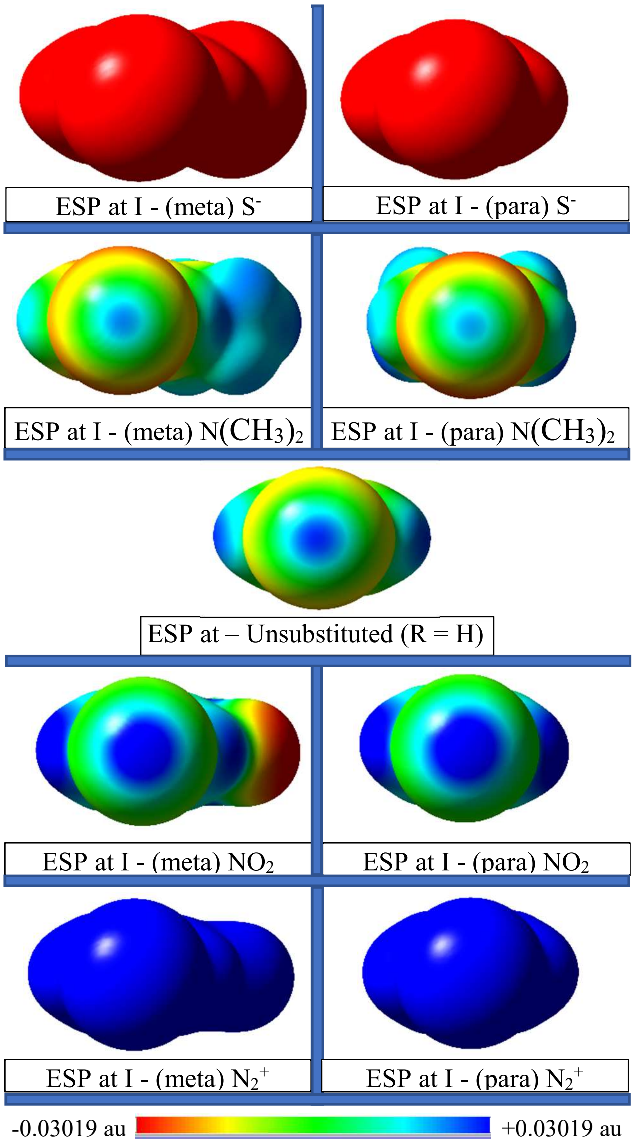

Figure S2: ESP maps showing the sigma hole on I (on the 0.001 au isodensity surface, all on the same scale: $\pm 3.019 \times 10^{-2}$ au.) for R = H and four other inductively distinct substituents (R = S^-, $N(CH_3)_2$, NO_2, and N_2^+) at both *meta* and *para* positions in C_6H_4RI. These structures are identical to those shown in Figure S1 but _include shadows for depth perception_. All structures are oriented with the ring horizontal and with I pointing out of the plane of the page. In the *meta* substituted species (on the left of this figure), the substituent is on the right of the ring.

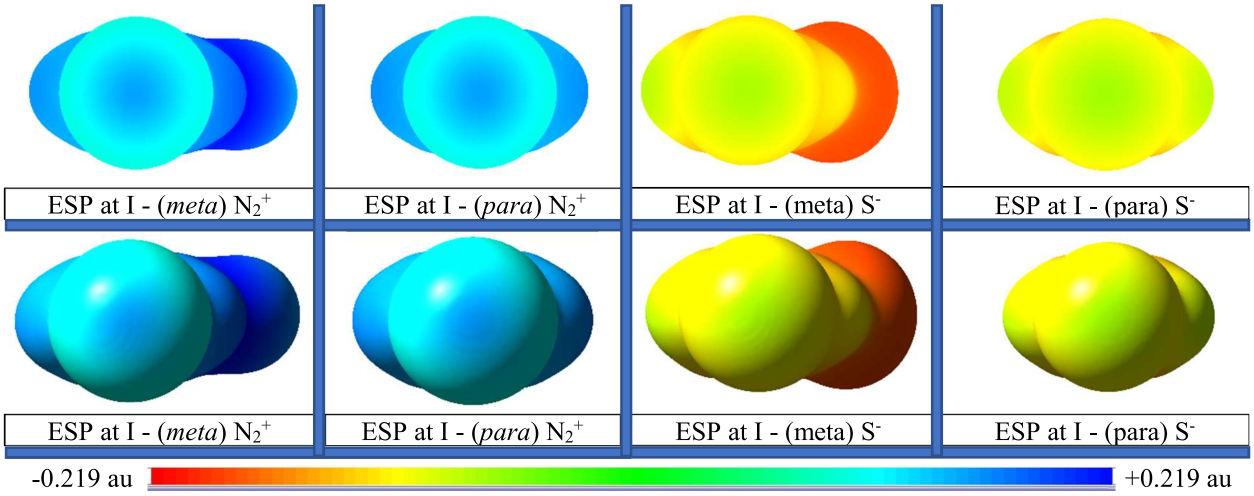

Figure S3: Electrostatic potential (ESP) maps without (top) and with (bottom) shadows for depth perception for two inductively distinct substituents, R = N_2^+, and S^-, at both *meta* and *para* positions in C_6H_4RI. Each figure shows the sigma hole on I (on the 0.001 au isodensity surface, all with the common ESP range: ± 0.219 au). All structures are oriented with the ring horizontal and with I pointing out of the plane. For *meta* cases, the substituent is on the right of the ring.

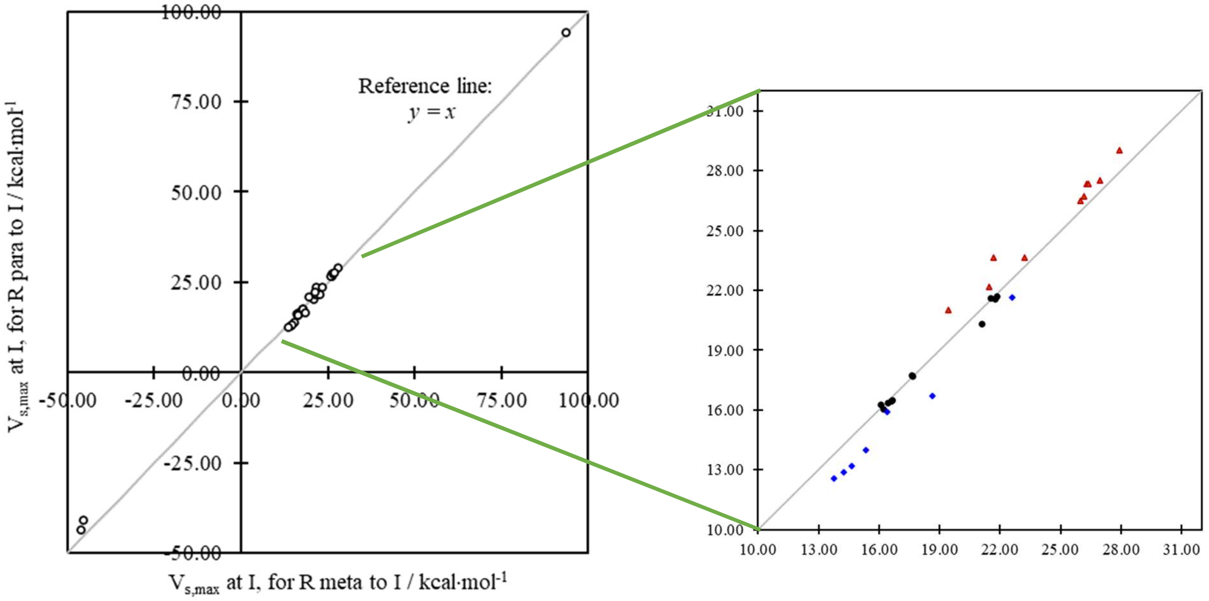

Figure S4: $V^{benz}_{(I+M);s,max}$: Plot of computed electrostatic potential (ESP) maxima at the sigma hole, in kcal·mol^{-1} units, on I (on the 0.001 au isodensity surface) of the R-C_6H_4-I substituted benzene ring in the gas phase – including the three charged species. A magnification of the compressed section with uncharged cases is shown. The actual values are in **Tables 1 and S2**.

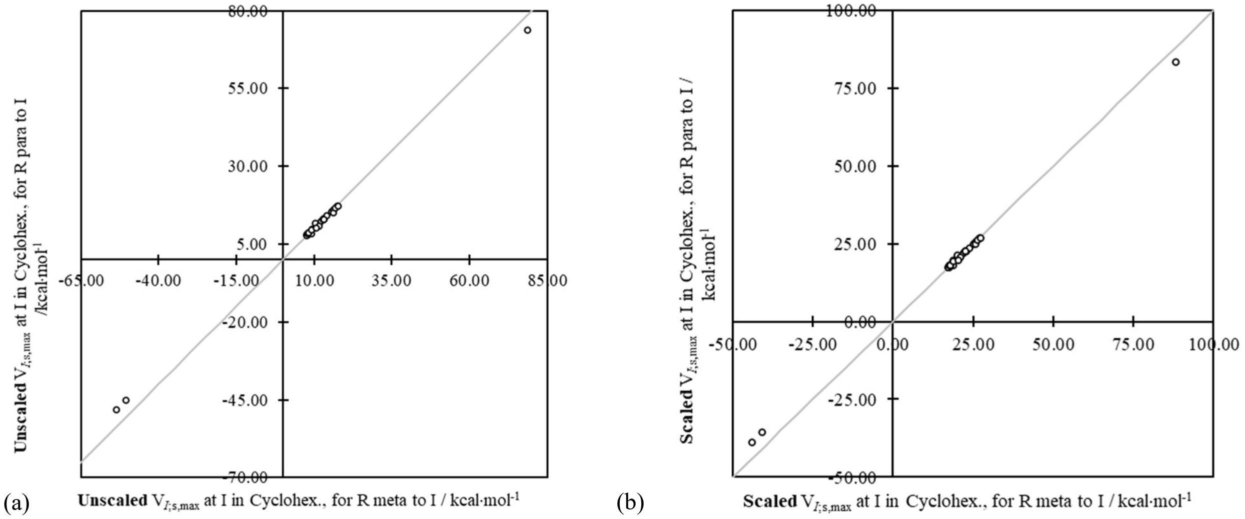

Figure S5: Plot of the computed (a) unscaled $V_{I;s,\max}^{cycl}$(R), and (b) scaled $V_{I;s,\max}^{scaled}(R)$ electrostatic potential (ESP) maxima at the sigma hole on I in kcal·mol^{-1} units (on the 0.001 au isodensity surface) for the substituted cyclohexane, $RC_6H_{10}I$, ring in the gas phase – including three charged species. The actual values are in **Table S3-S4**. The scaled values (**Table S4**) are equal to those unscaled values plus a $\Delta V_{s,max}$, which is equal to $V_{(I+M);s,max}^{benz}(\boldsymbol{H}) - V_{I;s,\max}^{cycl}(\mathbf{H})$.

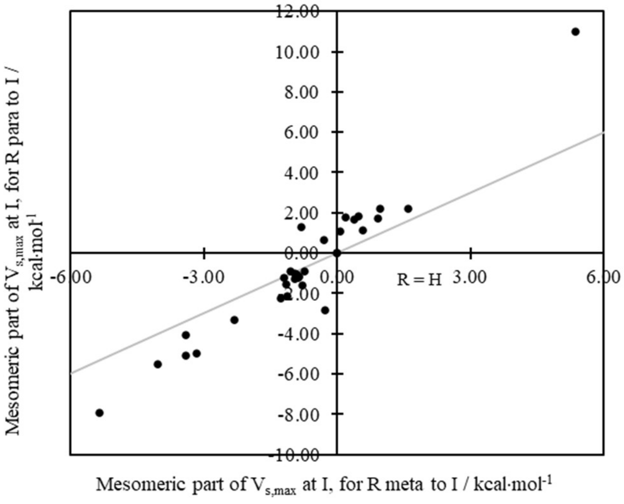

Figure S6: $V_{M;s,max}^{benz}(\boldsymbol{R})$: (**gas phase**) ESP contributions associated with π effects after scaled inductive components are removed from $V_{I+M;s,\max}^{benz}(R)$. See values in **Table 4** in the main text. This graph, which is also discussed in the main text, is included here for completeness. All of the $V_{M;s,max}^{benz}$ values are comfortably accommodated in this figure without compression, including the values for the charged cases. Similar plots are obtained for the implicit (**ethanol** and **water**) solvent cases. The relevant data for those cases are included in **Table 4** in the main text.

Supporting Notes

Supporting Note 1. On the Consistency of Computed Potentials for Different Substitution Patterns on Cyclohexane: To address the question of whether this outcome is an accident of our selection of the equatorial-equatorial (i.e. R_{eq}-I_{eq}) positions for I and R on the cyclohexane ring, we computed $V_{s,max}$ values for four different structural arrangements (R_{eq}-I_{eq} and R_{eq}-I_{ax}, both with R at the *meta* and *para* positions) for R = H, F, Cl, Br, I, NH_2, and CN. This subset of R groups was chosen in order to span the range of neutral donor and acceptor species considered (Figure S7).

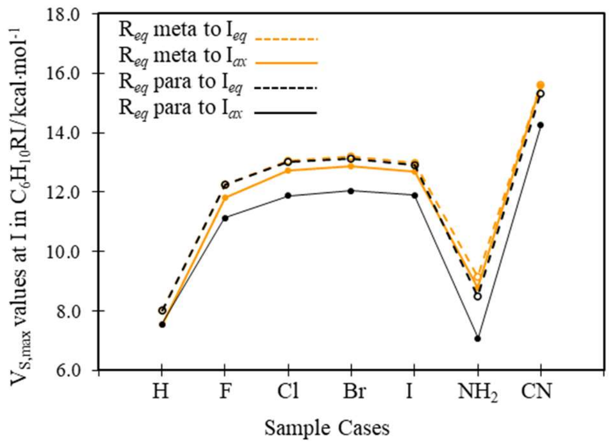

Figure S7: $V_{s,max}$ *values for a diverse set of R substituents and* $C_6H_{10}RI$ *chair isomers.*

This survey allowed us to assess the (in)variability of the potentials at I, $V_{I;s,\max}^{cycl}(R)$, as a function of the relative positions of R and I in $C_6H_{10}RI$. Cases with R in the axial position were excluded in order to avoid any possible escalation of steric effects (between bulky R groups and other parts of the ring), and we considered as well that the equatorial C-R bond in $C_6H_{10}RI$, pointing as it does away from the ring, is more consistent with the arrangement of the C-R bond in C_6H_4RI.

The results (Figure S7) show that the trend is the same in all cases. The actual values differ somewhat but by roughly constant amounts among the various configurations considered. For R = H, of course, the equatorial and axial cases are distinct isomers, but the *meta* and *para* forms of each are identical. The reason for the relatively low $V_{s,max}$ values in the *para* R_{eq}-I_{ax} case is unclear. But, even in that case, the overall trend going from one substituent to another remains essentially the same, which lends support to the assumption that the trend in inductive effects for R on I in the substituted cyclohexane systems is roughly independent of the axial or equatorial position of the I substituent on the ring. So, the general qualitative ordering of the substituents in terms of their inductive tendencies, and the consequences for the sigma hole potential at the terminal I center, is not an accident of the $C_6H_{10}RI$ configuration. It reflects, we find, the nature of the particular R substituent.

Tables

Table S1: Experiment based Hammett-type substituent constants, primarily from the list assembled by Hansch, Corwin, and Taft from various sources.†‡

R	σ_{meta}	σ_{para}	R	σ_{meta}	σ_{para}	R	σ_{meta}	σ_{para}
H	0		$B(OH)_3^-$	-0.48	-0.44	CN	0.56	0.66
F	0.34	0.06	S^-	-0.36	-1.21	CF_3	0.43	0.54
Cl	0.37	0.23	NHBut	-0.34	-0.51	COOH	0.37	0.45
Br	0.39	0.23	NH_2	-0.16	-0.66	$COOCH_3$	0.37	0.45
I	0.35	0.18	$NHCH_3$	-0.21	-0.70	NO_2	0.71	0.78
CH_3	-0.07	-0.17	NMe_2	-0.16	-0.83	SO_3H	0.38	0.64
CH_2CH_3	-0.07	-0.15	NHCHO	0.19	0.00	SO_2Cl	1.2	1.11
n-Pr	-0.06	-0.13	OH	0.12	-0.37	IF_4	1.07	1.15
i-Pr	-0.04	-0.15	OCH_3	0.12	-0.27	ICl_2	1.1	1.11
t-But	-0.10	-0.20	CHO	0.35	0.42	$N{\equiv}N^+$	1.76	1.91
Ph	0.06	-0.01						

†Hansch, Corwin.; Leo, A.; Taft, R. W. A Survey of Hammett Substituent Constants and Resonance and Field Parameters. *Chem. Rev.* **1991**, *91*, 165–195. (Table 1 in this reference)

‡ Imaizumi, H.; Koyanagi, T.; Zhao, D. Reactivity of Sulfonic Acid Group and Estimation of its Substituent-Effect in T-for-H Exchange Reaction *J. Radioanal. Nucl. Chem.,* **2002**, *252*, 467–472. The values for SO_3H are estimates from this reference: $\sigma_m = 0.38$, and $\sigma_p = 0.64$.

Table S2: Computed $V^{benz}_{(I+M);s,max}$ electrostatic potential (ESP) maxima at the sigma hole on I, in kcal·mol^{-1} units, on I (on the 0.001 au isodensity surface) for the R-C_6H_4-I ring in the gas phase (vacuum).

R	*meta*	*para*	R	*meta*	*para*	R	*meta*	*para*
H	17.67		$B(OH)_3^-$	-45.29	-40.93	CN	26.15	26.71
F	21.10	20.30	S^-	-46.18	-43.47	CF_3	23.22	23.66
Cl	21.76	21.54	NHBut	14.24	12.89	COOH	21.46	22.19
Br	21.86	21.70	NH_2	15.37	14.01	$COOCH_3$	19.46	21.02
I	21.53	21.60	$NHCH_3$	14.66	13.17	NO_2	26.29	27.37
CH_3	16.68	16.49	NMe_2	13.75	12.59	SO_3H	25.98	26.52
CH_2CH_3	16.61	16.42	NHCHO	22.60	21.63	SO_2Cl	27.91	29.05
n-Pr	16.24	16.04	OH	18.65	16.73	IF_4	26.40	27.35
i-Pr	16.46	16.35	OCH_3	16.39	15.92	ICl_2	26.97	27.54
t-But	16.11	16.25	CHO	21.70	23.65	$N{\equiv}N^+$	93.68	94.27
Ph	17.64	17.75						

Table S3: Computed **unscaled** ($V_{I;s,\max}^{cycl}$(**R**)) electrostatic potential (ESP) maxima at the sigma hole in kcal·mol^{-1} units on I (on the 0.001 au isodensity surface) of the R-C_6H_{10}-I substituted cyclohexane ring in the gas phase.

R	*meta*	*para*	R	*meta*	*para*	R	*meta*	*para*
H	8.05		$B(OH)_3^-$	-53.65	-48.30	CN	15.62	15.35
F	12.25	12.27	S^-	-50.45	-45.15	CF_3	13.04	12.94
Cl	13.05	13.00	NHBut	8.03	8.36	COOH	11.46	10.89
Br	13.20	13.10	NH_2	9.16	8.48	$COOCH_3$	10.65	10.12
I	12.95	12.89	$NHCH_3$	8.20	8.54	NO_2	16.20	15.93
CH_3	8.01	8.13	NMe_2	8.16	8.51	SO_3H	16.17	15.12
CH_2CH_3	7.84	7.97	NHCHO	14.09	14.13	SO_2Cl	17.34	17.25
n-Pr	7.77	7.95	OH	9.31	9.96	IF_4	16.71	16.64
i-Pr	7.71	7.94	OCH_3	9.09	9.62	ICl_2	17.64	17.28
t-But	7.68	7.86	CHO	10.49	11.81	$N{\equiv}N^+$	78.70	73.67
Ph	8.76	9.03						

The electrostatic potentials were generated on the 0.001au surfaces. For R = I, the values are typically identical on both I centers. If they differ in any marginal way, the average values are used.

Table S4: **Scaled Inductive Component:** $V_{I;s,\max}^{scaled}(R)$ obtained from $V_{I;s,\max}^{cycl}$(**R**) plus $\Delta V^{benz\text{-}cycl}$**(H)** for the gas phase

R	*meta*	*para*	R	*meta*	*para*	R	*meta*	*para*
H	17.67		$B(OH)_3^-$	-44.04	-38.68	CN	25.24	24.96
F	21.87	21.88	S^-	-40.83	-35.54	CF_3	22.66	22.56
Cl	22.67	22.61	NHBut	17.64	17.98	COOH	21.07	20.51
Br	22.82	22.72	NH_2	18.78	18.10	$COOCH_3$	20.27	19.73
I	22.57	22.51	$NHCH_3$	17.82	18.15	NO_2	25.82	25.54
CH_3	17.63	17.75	NMe_2	17.78	18.13	SO_3H	25.79	24.74
CH_2CH_3	17.45	17.59	NHCHO	23.71	23.75	SO_2Cl	26.95	26.86
n-Pr	17.39	17.57	OH	18.93	19.58	IF_4	26.32	26.26
i-Pr	17.33	17.56	OCH_3	18.70	19.23	ICl_2	27.26	26.90
t-But	17.30	17.48	CHO	20.10	21.42	$N{\equiv}N^+$	88.31	83.29
Ph	18.37	18.64						

Table S5: **Mesomeric Component:** $V_{M;s,\max}^{benz}(R)$ obtained from $V_{(I+M);s,max}^{benz}(R)$ and $V_{I;s,\max}^{scaled}(R)$ for the gas phase

R	*meta*	*para*	R	*meta*	*para*	R	*meta*	*para*
H	0.00		$B(OH)_3^-$	-1.26	-2.25	CN	0.91	1.74
F	-0.78	-1.59	S^-	-5.35	-7.93	CF_3	0.57	1.11
Cl	-0.91	-1.07	NHBut	-3.40	-5.09	COOH	0.38	1.68
Br	-0.96	-1.02	NH_2	-3.41	-4.09	$COOCH_3$	-0.81	1.29
I	-1.04	-0.91	$NHCH_3$	-3.16	-4.98	NO_2	0.47	1.82
CH_3	-0.95	-1.27	NMe_2	-4.03	-5.54	SO_3H	0.20	1.77
CH_2CH_3	-0.85	-1.17	NHCHO	-1.11	-2.11	SO_2Cl	0.96	2.19
n-Pr	-1.15	-1.53	OH	-0.28	-2.85	IF_4	0.08	1.09
i-Pr	-0.87	-1.21	OCH_3	-2.32	-3.31	ICl_2	-0.29	0.64
t-But	-1.19	-1.23	CHO	1.59	2.23	$N{\equiv}N^+$	5.37	10.98
Ph	-0.73	-0.90						

Table S6: Computed **unscaled** ($V_{I;s,\max}^{cycl}(\mathbf{R})$) electrostatic potential (ESP) maxima in kcal·mol^{-1} units for the sigma hole on I (on the 0.001 au isodensity surface) of the R-C_6H_{10}-I substituted cyclohexane ring and **Scaled Inductive Component:** $V_{I;s,\max}^{scaled}(R)$ obtained from $V_{I;s,\max}^{cycl}(\mathbf{R})$ plus $\Delta V^{benz\text{-}cycl}(\mathbf{H})$ for the gas phase (vacuum), and implicit solvent environments for ethanol and water.

	Unscaled						Scaled					
	Vacuum		Ethanol		Water		Vacuum		Ethanol		Water	
R	***meta*-R**	***para*-R**	***meta*-R**	***para*-R**	***meta*-R**	***para*-R**	***meta*-R**	***para*-R**	***meta*-R**	***para*-R**	***meta*-R**	***para*-R**
H	8.05		2.88		2.63		17.67		16.70		16.69	
F	12.25	12.27	8.18	8.31	7.96	8.11	21.87	21.88	21.99	22.13	22.02	22.17
Cl	13.05	13.00	9.23	9.26	9.02	9.06	22.67	22.61	23.05	23.08	23.08	23.13
Br	13.20	13.10	9.42	9.38	9.21	9.18	22.82	22.72	23.24	23.20	23.27	23.24
I	12.95	12.89	9.03	9.01	8.80	8.80	22.57	22.51	22.85	22.83	22.87	22.86
CH_3	8.01	8.13	2.90	3.05	2.65	2.80	17.63	17.75	16.72	16.87	16.71	16.86
CH_2CH_3	7.84	7.97	2.73	2.93	2.48	2.68	17.45	17.59	16.55	16.75	16.54	16.75
n-Pr	7.77	7.95	2.65	2.92	2.40	2.68	17.39	17.57	16.47	16.74	16.46	16.74
i-Pr	7.71	7.94	2.56	2.87	2.31	2.62	17.33	17.56	16.38	16.69	16.37	16.68
t-But	7.68	7.86	2.59	2.80	2.34	2.55	17.30	17.48	16.41	16.62	16.40	16.62
Ph	8.76	9.03	4.17	4.56	3.94	4.34	18.37	18.64	17.98	18.38	18.00	18.40
$B(OH)_3^-$	-53.65	-48.30	-44.99	-38.60	-44.59	-38.24	-44.04	-38.68	-31.17	-24.78	-30.53	-24.18
S^-	-50.45	-45.15	-41.54	-35.24	-41.20	-34.83	-40.83	-35.54	-27.72	-21.42	-27.14	-20.77
NHBut	8.03	8.36	3.26	3.91	3.02	3.70	17.64	17.98	17.08	17.73	17.08	17.76
NH_2	9.16	8.48	4.70	3.96	4.49	3.75	18.78	18.10	18.52	17.77	18.55	17.81
$NHCH_3$	8.20	8.54	3.19	3.98	2.93	3.78	17.82	18.15	17.00	17.80	17.00	17.84
NMe_2	8.16	8.51	3.25	3.99	3.00	3.78	17.78	18.13	17.07	17.81	17.07	17.84
NHCHO	14.09	14.13	9.50	9.58	9.23	9.31	23.71	23.75	23.31	23.40	23.29	23.37
OH	9.31	9.96	4.53	5.62	4.29	5.41	18.93	19.58	18.35	19.44	18.35	19.47
OCH_3	9.09	9.62	4.46	5.43	4.22	5.24	18.70	19.23	18.28	19.25	18.28	19.30
CHO	10.49	11.81	5.09	7.26	4.80	7.03	20.10	21.42	18.91	21.08	18.86	21.09
CN	15.62	15.35	11.49	11.19	11.25	10.95	25.24	24.96	25.31	25.01	25.31	25.01
CF_3	13.04	12.94	8.70	8.62	8.46	8.39	22.66	22.56	22.52	22.44	22.52	22.45
COOH	11.46	10.89	7.34	6.56	7.12	6.34	21.07	20.51	21.15	20.37	21.19	20.40
$COOCH_3$	10.65	10.12	6.68	5.93	6.47	5.73	20.27	19.73	20.49	19.75	20.53	19.79
NO_2	16.20	15.93	12.22	12.05	11.98	11.83	25.82	25.54	26.04	25.87	26.05	25.89
SO_3H	16.17	15.12	13.77	11.74	13.68	11.57	25.79	24.74	27.58	25.56	27.74	25.63
SO_2Cl	17.34	17.25	13.96	13.91	13.74	13.70	26.95	26.86	27.78	27.73	27.80	27.77
IF_4	16.71	16.64	13.16	13.38	12.94	13.15	26.32	26.26	26.98	27.16	27.00	27.21
ICl_2	17.64	17.28	14.04	13.82	13.80	13.60	27.26	26.90	27.85	27.64	27.86	27.66
$N{\equiv}N^+$	78.70	73.67	63.19	56.82	62.46	56.01	88.31	83.29	77.01	70.63	76.52	70.07

Set of Graphs for Alternative Scaling with $V_{s,max}$(H) = 0.

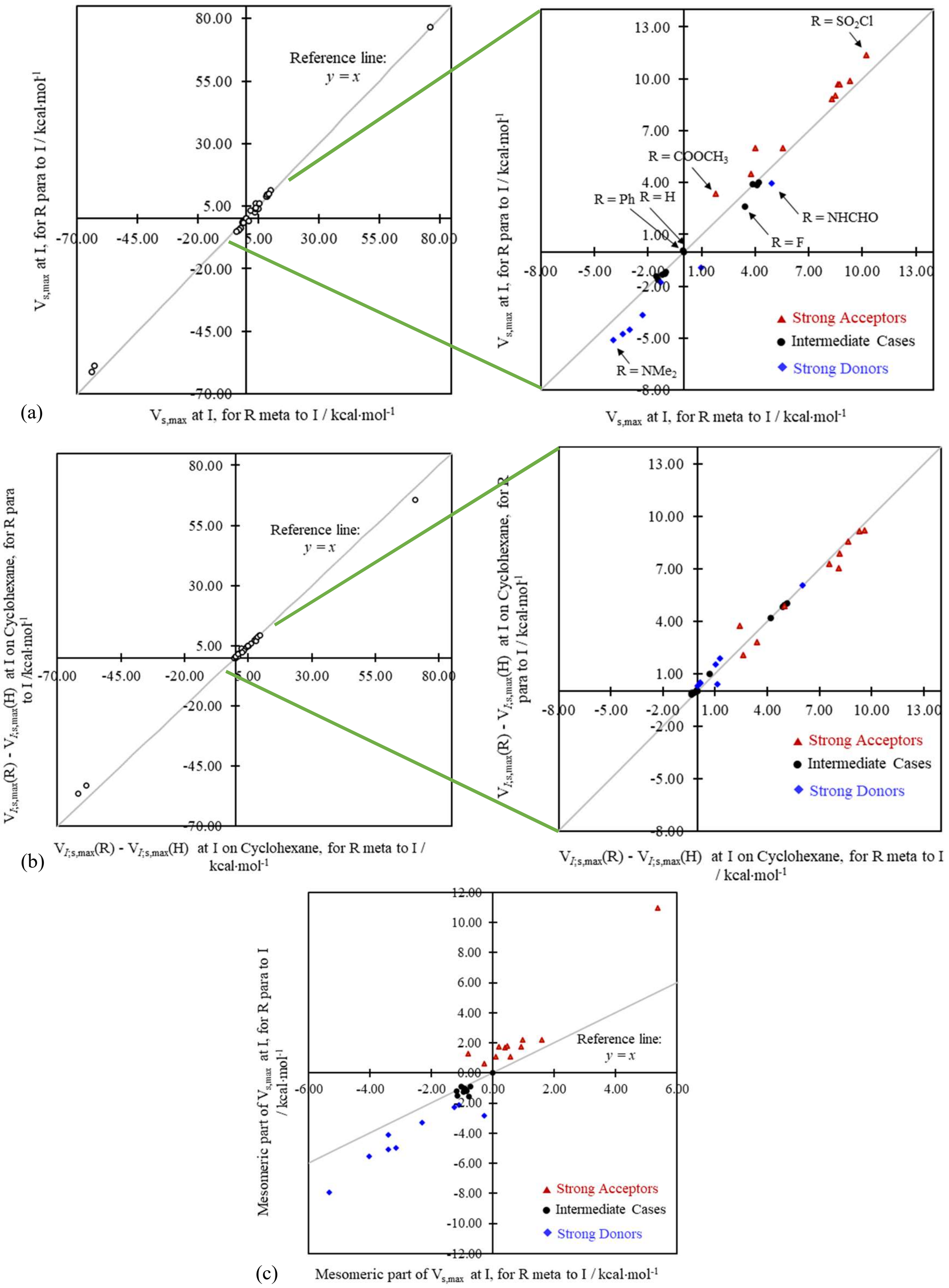

Figure S8: *Graphs of computed (gas phase) potentials from I on substituted benzene (a) and cyclohexane (b) molecules adjusted so $V_{s,max}(H) = 0$, magnifying the region with the bulk of the data, and the mesomeric component (c). For the latter, no magnification is necessary and the graph, (c), is identical to Figure S6. The corresponding values are shown in the main text.*

Full Gaussian 16 Reference

Gaussian 16, Revision B.01, Frisch, M. J.; Trucks, G. W.; Schlegel, H. B.; Scuseria, G. E.; Robb, M. A.; Cheeseman, J. R.; Scalmani, G.; Barone, V.; Petersson, G. A.; Nakatsuji, H.; Li, X.; Caricato, M.; Marenich, A. V.; Bloino, J.; Janesko, B. G.; Gomperts, R.; Mennucci, B.; Hratchian, H. P.; Ortiz, J. V.; Izmaylov, A. F.; Sonnenberg, J. L.; Williams-Young, D.; Ding, F.; Lipparini, F.; Egidi, F.; Goings, J.; Peng, B.; Petrone, A.; Henderson, T.; Ranasinghe, D.; Zakrzewski, V. G.; Gao, J.; Rega, N.; Zheng, G.; Liang, W.; Hada, M.; Ehara, M.; Toyota, K.; Fukuda, R.; Hasegawa, J.; Ishida, M.; Nakajima, T.; Honda, Y.; Kitao, O.; Nakai, H.; Vreven, T.; Throssell, K.; Montgomery, J. A., Jr.; Peralta, J. E.; Ogliaro, F.; Bearpark, M. J.; Heyd, J. J.; Brothers, E. N.; Kudin, K. N.; Staroverov, V. N.; Keith, T. A.; Kobayashi, R.; Normand, J.; Raghavachari, K.; Rendell, A. P.; Burant, J. C.; Iyengar, S. S.; Tomasi, J.; Cossi, M.; Millam, J. M.; Klene, M.; Adamo, C.; Cammi, R.; Ochterski, J. W.; Martin, R. L.; Morokuma, K.; Farkas, O.; Foresman, J. B.; Fox, D. J. Gaussian, Inc., Wallingford CT, 2016.
